# DFT Calculations of ^1^H NMR Chemical Shifts of Geometric Isomers of Conjugated Linolenic Acids, Hexadecatrienyl Pheromones, and Model Triene-Containing Compounds: Structures in Solution and Revision of NMR Assignments

**DOI:** 10.3390/molecules26113477

**Published:** 2021-06-07

**Authors:** Themistoklis Venianakis, Christina Oikonomaki, Michael G. Siskos, Alexandra Primikyri, Ioannis P. Gerothanassis

**Affiliations:** Section of Organic Chemistry and Biochemistry, Department of Chemistry, University of Ioannina, GR-45110 Ioannina, Greece; t.venianakis@uoi.gr (T.V.); pch01328@uoi.gr (C.O.); a.primikyri@uoi.gr (A.P.)

**Keywords:** CLnAs, hexadecatrienyl pheromones, chemical shifts, DFT, GIAO, NMR

## Abstract

A DFT study of the ^1^H NMR chemical shifts, δ(^1^H), of geometric isomers of 18:3 conjugated linolenic acids (CLnAs), hexadecatrienyl pheromones, and model triene-containing compounds is presented, using standard functionals (B3LYP and PBE0) as well as corrections for dispersion interactions (B3LYP-D3, APFD, M06–2X and ωB97XD). The results are compared with literature experimental δ(^1^H) data in solution. The closely spaced “inside” olefinic protons are significantly more deshielded due to short-range through-space H^…^H steric interactions and appear close to or even beyond δ-values of aromatic systems. Several regularities of the computational δ(^1^H) of the olefinic protons of the conjugated double bonds are reproduced very accurately for the lowest-energy DFT-optimized single conformer for all functionals used and are in very good agreement with experimental δ(^1^H) in solution. Examples are provided of literature studies in which experimental resonance assignments deviate significantly from DFT predictions and, thus, should be revised. We conclude that DFT calculations of ^1^H chemical shifts of trienyl compounds are powerful tools (i) for the accurate prediction of δ(^1^H) even with less demanding functionals and basis sets; (ii) for the unequivocal identification of geometric isomerism of conjugated trienyl systems that occur in nature; (iii) for tackling complex problems of experimental resonance assignments due to extensive signal overlap; and (iv) for structure elucidation in solution.

## 1. Introduction

**Figure 1:**
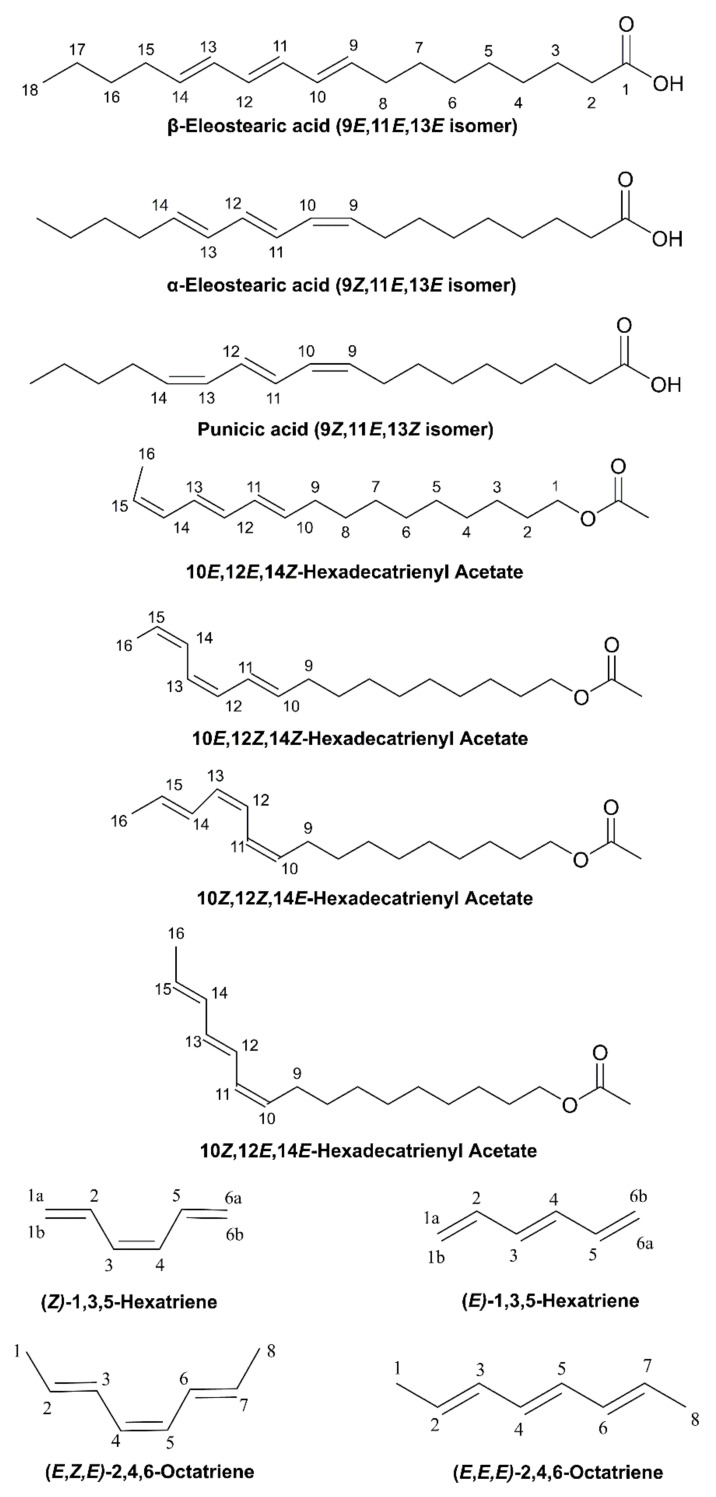
Chemical structures of three geometric isomers of the 18:3 ω-5 conjugated linolenic acid, four geometric isomers of the 16:3 ω-2 conjugated hexadecatrienyl acetate, and triene-containing model compounds investigated in the present work.

Conjugated linolenic acids (CLnAs) are a group of positional and geometric isomers of octadecatrienoic acids (C18:3) that contain three conjugated double bonds primarily in positions Δ9,11,13 and Δ8,10,12 [1,2]. α-Eleostearic acid (α-ESA) has three conjugated double bonds (C18:3Δ9 *cis*, 11 *trans*, 13 *trans*) (Figure 1) and it is produced and stored in the seed oil of plants such as *A. fordii*, *M. charantia*, *Parinarium* spp., and *P. mahaleb*. α-ESA was reported to have anticancer properties on several tumor cell lines, such as A549 (lung), MCF-7 (breast), DLD1 (colorectal), MKN-7 (stomach), and HepG2 (hepatoma). Lipid peroxidation has been suggested as the underlying mechanism [3]. β-Eleostearic acid (β-ESA) (C18:3Δ9 *trans*, 11 *trans*, 13 *trans*) (Figure 1) is present in pomegranate, bitter gourd, and *catalpa* [4]. β-ESA was reported to have a stronger antiproliferative effect than the geometric isomer α-ESA. A decrease in Bcl-2 and an increase in Bax mRNA expression along with DNA fragmentation were observed, which indicates different signaling pathways than their *cis* isomers [5]. Punicic acid (PA) (C18:3Δ9 *cis*, 11 *trans*, 13 *cis*; Figure 1) is a fatty acid derived from the fruit of *P. granatum* (aspomegranate) and from *T. kirilowii*. PA has been reported to have several health benefits such as anticancer activity and the prevention of obesity and insulin resistance in mice [6,7,8]. The cytotoxic properties on human monocytic leukemia cells have been attributed to lipid peroxidation [9].

Molecules with three conjugated double bonds have also been identified in hexadecatrienyl systems (Figure 1) from several lepidopterous species [10]. These sex hormones from females attract male moths and, thus, have significant roles in forest ecology, human health, and communication biology [11]. Pheromone-baited traps, therefore, have been widely used for the detection, population monitoring, and control of pest species and for assessing the efficacy of eradication efforts [12]. Isolation and identification of sex pheromones, however, is a difficult task due to the very small amount of pheromone produced by the insects and the small number of individuals available for the analysis of the pheromone gland content [12]. Initially, the isomeric configuration of the natural trienals was unknown and their identity was investigated by synthesis and the use of NMR spectroscopy [13,14,15]. Several geometric isomers, however, are not amenable to complete resonance assignment due to extensive signal overlap.

Hexadecatrienyl pheromones and their synthetic analogues [11,13,14,15], conjugated linolenic acids (CLnAs) [16,17,18,19,20], and model triene-containing compounds [21,22,23,24] have been extensively investigated using ^1^H and ^13^C NMR. 2D-NOESY experiments, ^13^C–^1^H COSY correlations, and analysis of spin–spin coupling constants with the use of homonuclear decoupling techniques were used to identify the geometric configurations of the trienyl conjugated double bonds. However, in several cases, severe overlap in the NMR spectra leads to equivocal signal assignments even when using 2D spectra and, consequently, to ambiguities in the spectral interpretations.

Several studies have been published that combine experimental NMR chemical shifts with computations for tackling the complex problems of resonance assignment, resonance reassignment, and structural revision [25,26,27,28,29,30,31,32,33,34,35,36,37] and for investigating high-resolution structures in solution [38,39,40,41,42,43,44,45,46,47]. DFT calculations of NMR chemical shifts in conjugated systems, however, are limited to ^1^H and ^13^C NMR chemical shifts of retinal isomers [48], and geometric isomers of diene-containing compounds [47]. Since no X-ray structures of conjugated linolenic acids (CLnAs) and hexadecatrienyl pheromones have so far been published, it would be of interest to use quantum chemical calculations of δ(^1^Η) for structure elucidation in solution. In this paper, we discuss (i) the effect of various functionals and basis sets on the accuracy of the DFT calculation of ^1^H NMR chemical shifts using the GIAO [49] technique on several geometric conjugated linolenic acids, hexadecatrienyl pheromones, and model triene-containing compounds (Figure 1); (ii) the use of δ(^1^H) for the unequivocal assignment of geometric isomerism and revision of literature experimental assignments; and (iii) the use of δ(^1^H) as a tool for structure elucidation in solution.

## 2. Results and Discussion

### 2.1. DFT-Calculated vs. Experimental ^1^H NMR Chemical Shifts of Model Compounds in Solution: Effects of Various Functionals and Basis Sets

**Figure 2:**
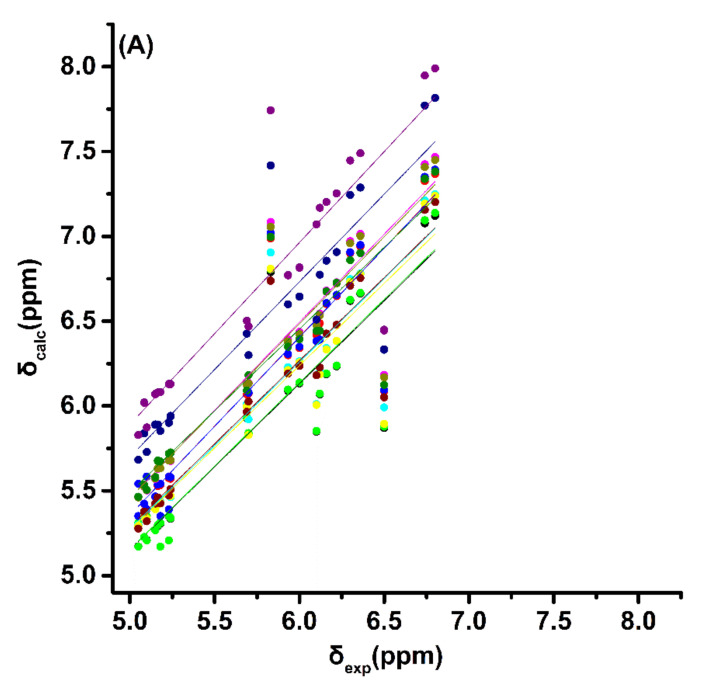

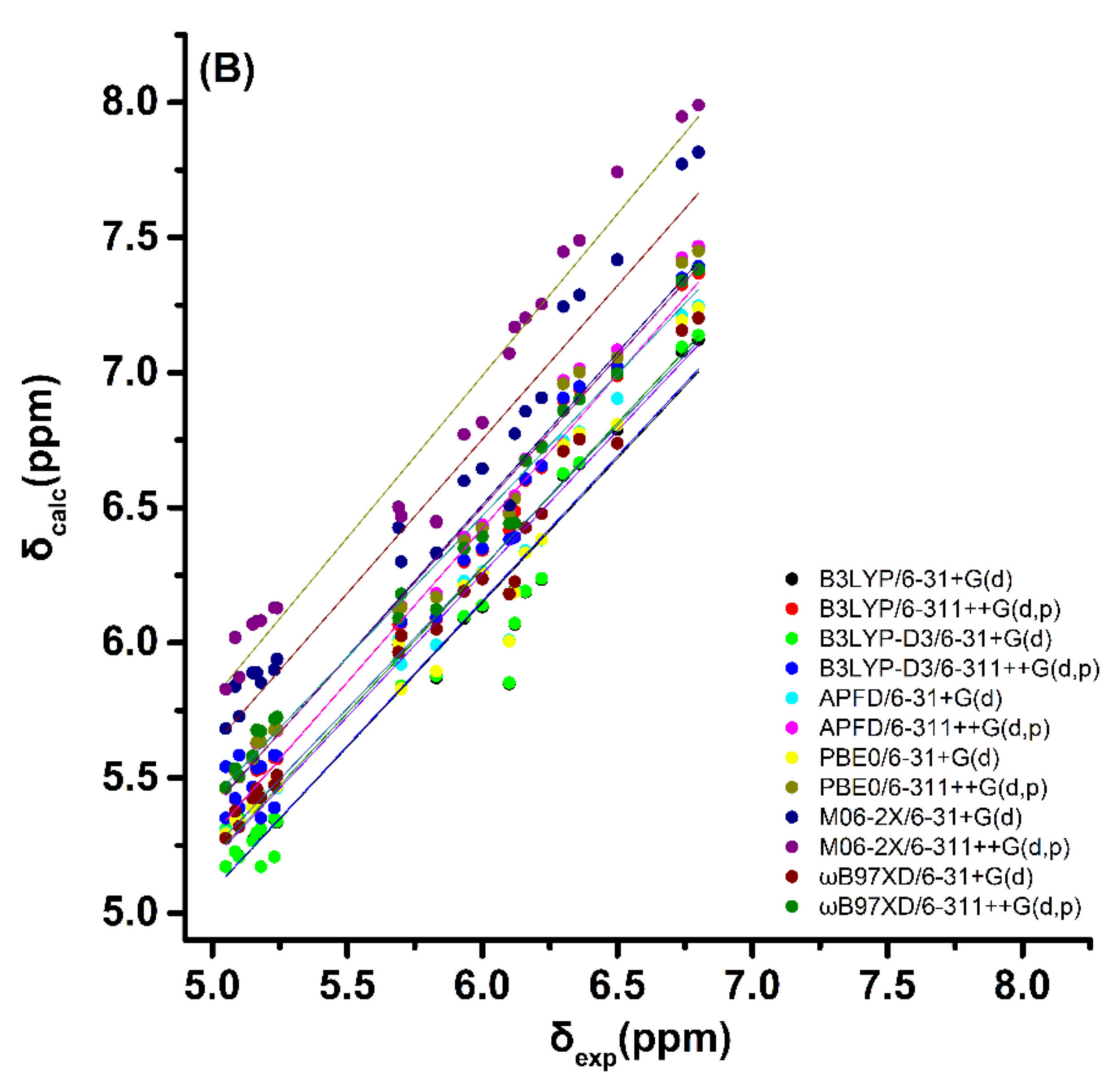
(**A**) Calculated, δ_calc_, ^1^H NMR chemical shifts with CPCM of the olefinic protons vs. experimental, δ_exp_, chemical shifts using the same level of theory as geometry optimization for the model trienyl compounds (*Z*)-1,3,5-hexatriene, (*E*)-1,3,5-hexatriene, (*E,Z,E*)-2,4,6-octatriene, and (*E,E,E*)-2,4,6-octatriene. (**B**) The same as in (A), but the literature experimental values of δ(H (3,6)) = 5.83 ppm and δ(H (4,5)) = 6.50 ppm of (*E,Z,E*)-2,4,6-octatriene [23] have been reversed.

The experimental ^1^H NMR chemical shifts of the trienyl model compounds (*Z*)-1,3,5-hexatriene, (*E*)-1,3,5-hexatriene, (*E,Z,E*)-2,4,6-octatriene and (*E,E,E*)-2,4,6-octatriene (Figure 1 and Table 1) exhibit resonances of the olefinic protons of the conjugated double bonds in the range of 5.0 to 6.8 ppm. The =CH_2_ protons are shielded with respect to the rest of the olefinic protons and appear in a narrow range of 5.05 to 5.23 ppm. Alkyl substitution of the H_1a_ proton, as in the case of (*E,Z,E*)-2,4,6-octatriene, results in a deshielding of ~0.5 ppm, which is in excellent agreement with ^1^H additive contribution to ethylene of Δδ_gem_ = 0.45 ppm [50]. Interestingly, the closely spaced “inside” olefinic 2,5 protons of (*Z*)-1,3,5-hexatriene and 3,6 protons of (*E,Z,E*)-2,4,6-octatriene (Figure 1) are the most deshielded and appear close to or even beyond δ-values of aromatic systems. This deshielding effect can be attributed to rigid geometries and significant H^…^H van der Waals repulsive effects of specific protons [22]. It has been demonstrated that hydrogen atoms, which are subject to significant steric compression, exhibit a deshielding that is dependent on the geometrical relationship between the H–C bond and the interacting proximate hydrogen [51]. This occurs because the symmetry of the electron cloud about the proton is disturbed, thus resulting in reduced electronic charge and diamagnetic shielding [52,53]. Trienyl model compounds, therefore, are ideally suited for the study of steric effects on δ(^1^H) using various functionals and basis sets. Energy minimization of the structures was performed with standard functionals (B3LYP and PBE0) as well as with corrections for dispersion interactions (B3LYP-D3, APFD, M06–2X, and ωB97XD). Inclusion of nonlocal van der Waals density functionals has been shown to improve the accuracy of standard DFT functionals [54,55]. The 6–31+G(d) and 6–311++G(d,p) basis sets were used to compare the results of a low computational cost basis set with a medium-size one. Computations of δ_calc_(^1^H) were performed (a) using a single methodology at the GIAO/B3LYP/6–311+G(2d, p)/CPCM level (Figure S1 and Figure S2 and Table S1), as recommended in [28], and (b) using the same level of theory as geometry optimization (Figure 2, Figure S3, and Table S2).

For all the functionals and basis sets used, the literature experimental chemical shifts of the closely spaced “inside” olefinic protons with δ(H (3,6)) = 5.83 ppm and δ(H (4,5)) = 6.50 ppm of (*E,Z,E*)-2,4,6-octatriene [23] strongly deviate from linearity (Figure S2A and Figure S3A). Revision of the literature experimental chemical shift data so that the inner H(3,6) protons to be deshielded to a larger degree than H(4,5) (Figure S2B and Figure S3B) results in very significant improvements of the regression coefficients and standard deviations for all the functionals and basis sets used (Table S3 and Table S4). The use of several DFT functionals with distinct contributions (long-range corrections or dispersion interactions) would provide distinct results for the geometry. The use, however, of a single methodology at the GIAO/B3LYP/6–311+G(2d,p) level for chemical shift computations results in identical correlations coefficients and mean square errors for all the functionals and basis sets used (Table S3). The use of the same level of theory in δ_calc_ (^1^H) as geometry optimization results in similar but not identical statistical data (Table S4). The quality of the linear regression procedure is a criterion whether the computational method is able to reproduce the experimental chemical shifts free from random error [28]. Furthermore, the extent to which the slope of the correlation line deviates from unity and the intercept from zero is a measure of the overall systematic error. Among the functionals with corrections for dispersion interactions, the ωB97XD with the 6–31+G(d) basis set performs better (intercept: 0.003, slope: 1.046; Table S4). The M06–2X functional is less suitable for shielding calculations of the selected molecules in agreement with literature data on several small molecules [56].

From the above, it can be concluded that the accuracy of the DFT calculations can provide a practical guide for future experimental assignments and help in the reassignment of literature experimental ^1^H NMR chemical shifts of triene-containing compounds based on a typical workflow that includes the following steps:

(i) the ^1^H NMR spectra are recorded in, e.g., a CDCl_3_ solution at 298 K, and a preliminary assignment is performed using a variety of 1D and 2D NMR experiments;
(ii) the ^1^H NMR chemical shifts are computed with the CPCM model at the same level of theory as geometry optimization or at the GIAO/B3LYP/6–311+G(2d,p) level, even with less demanding functionals and basis sets;
(iii) a very good linear correlation between experimental NMR chemical shifts, δ_exp_, and calculated shifts, δ_calc_, provides a strong indication that the assignment procedure is correct.

### 2.2. Out-of-Plane Deformation of the Trienyl System and Steric Effects in Model Compounds

**Figure 3:**
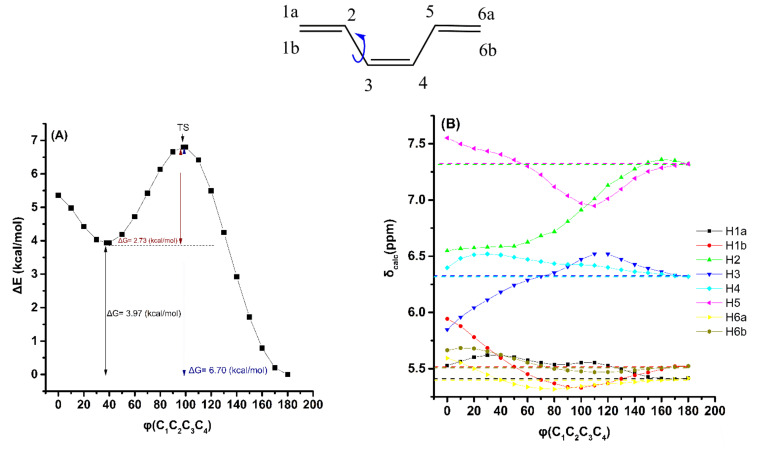
Effect of the variation in the torsion angle φ (C_1_C_2_C_3_C_4_) of (*Z*)-1,3,5-hexatriene, with energy minimization at the B3LYP/6–31+G(d) level, on the electronic energy Δ*E* (kcal/mol) (characteristic Δ*G* values are also shown) (**A**), and δ_calc_(^1^H) data of the olefinic protons (at the GIAO/B3LYP/6–311+G(2d,p) level with CPCM in CHCl_3_) (**B**). The experimental chemical shift values are denoted with the horizontal dotted lines.

Variation of the torsion angle φ(C_1_,C_2_,C_3_,C_4_) of the trienyl system of (*Z*)-1,3,5-hexatriene, in steps of 10° in the range of 180° to 0°, with energy minimization at the B3LYP/6–31+G(d) level, results in significant changes in the electronic energy (Δ*E*) (Figure 3A). A second minimum at φ = 40° was observed with Δ*G* = 2.37 kcal·mol^−1^ higher than that of the φ = 180° conformer. The significant Δ*G* energy difference of the two low-energy conformers with φ = 40° and φ = 180.0° shows that the δ_calc_(^1^H) data (at the GIAO/B3LYP/6–311+G(2d,p) level), weighted by the respective Boltzmann factor, are nearly identical with those of the φ = 180.0° conformer. The effect of the population of the φ = 40° conformer can, thus, be neglected. The effect of variation of the torsion angle φ on ^1^H NMR chemical shifts is shown in Figure 3B and Table S5. The δ_calc_(^1^H) data indicate similar behavior of the “inside” H2 and H5 protons with a pronounced shielding at 100° < φ < 180° (Figure 3B). In the range of torsion angles 0°< φ < 100°, the H5 proton shows a strong deshielding with a maximum value of δ = 7.32 ppm at φ = 0°. This clearly demonstrates that the strong, through-space, steric interaction with the H_1b_ is the primary factor that results in δ values in the range of aromatic protons. The effect of variation of the torsion angle φ of (*Z*)-1,3,5-hexatriene on calculated olefinic ^1^H NMR chemical shifts using the same level of theory as geometry optimization (APFD/6–311++G(d,p)) is shown in Figure S4 and Table S6. The results are very similar to those of Figure 3 and Table S5; however, the Δ*Ε* and Δ*G* values are slightly smaller and the deshielding effect is slightly more pronounced due to the dispersion interaction of the APFD functional.

**Figure 4:**
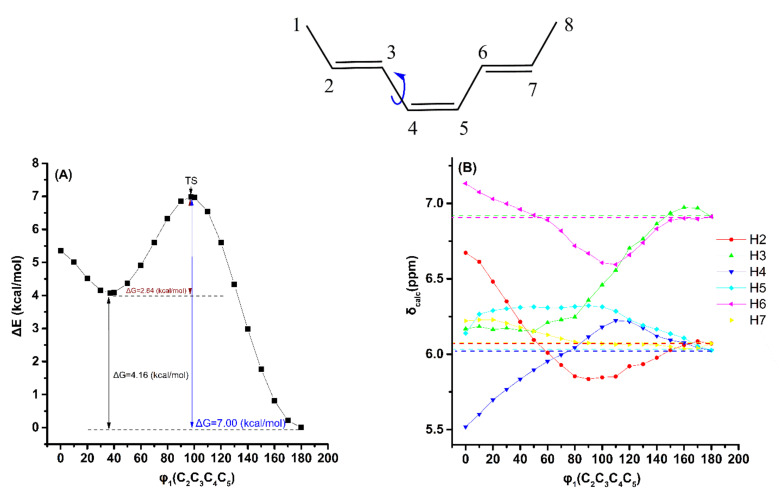
Effect of variation in the torsion angle φ_1_(C_2_C_3_C_4_C_5_) of (*E*,*Z,E*)-2,4,6-octatriene, with energy minimization at the B3LYP/6–31+G(d) level, on the electronic energy Δ*E* (kcal/mol) (characteristic Δ*G* values are also shown) (**A**) and on the olefinic δ_calc_(^1^H) data (at the GIAO/B3LYP/6–311+G(2d,p) level with CPCM in CHCl_3_) (**B**). The experimental chemical shift values are denoted with the horizontal dotted lines.

The electronic energy Δ*Ε* (kcal/mol) and shielding changes due to the variation in the torsion angle φ_1_ (C_2_C_3_C_4_C_5_) of the (*E,Z,E*)-2,4,6-octatriene (Figure 4, Table S7) are very similar to those due to the variation in the torsion angle φ of (*Z*)-1,3,5-hexatriene. Thus, the δ_calc_(^1^H) data of the low-energy conformers, weighted by the respective Boltzmann factor, are identical with those of the φ_1_ = 180° conformer. Again, the effect of variation of the φ_1_ torsion angle on δ_calc_(^1^H), using the same level of theory as geometry optimization (APFD/6–311++G(d,p)), is to decrease slightly the Δ*Ε* and Δ*G* values and increase the deshielding effect (Figure S5 and Table S8).

**Figure 5:**
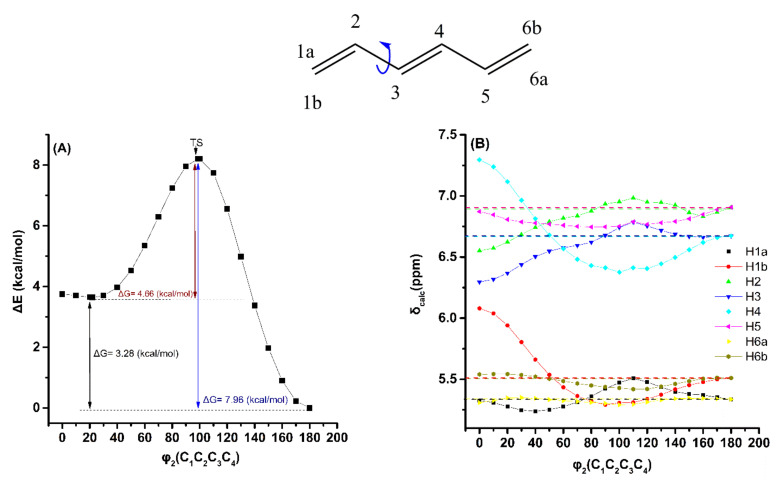
Effect of variation in the torsion angle φ_2_(C_1_C_2_C_3_C_4_) of (*E*)-1,3,5-hexatriene, with energy minimization at the B3LYP/6–31+G(d) level, on the electronic energy Δ*E* (kcal/mol) (characteristic Δ*G* values are also shown) (**A**) and on the olefinic δ_calc_(^1^H) data (at the GIAO/B3LYP/6–311+G(2d,p) level with CPCM in CHCl_3_) (**B**). The experimental chemical shift values are denoted with the horizontal dotted lines.

The effect of the variation in the torsion angle φ_2_(C_1_C_2_C_3_C_4_) of (*E*)-1,3,5-hexatriene, with energy minimization at the B3LYP/6–31+G(d) level, on the electronic energy Δ*Ε*(kcal·mol^−1^), and δ_calc_(^1^H), at the GIAO/B3LYP/6–311+G(2d,p) level, are shown in Figure 5. A second broad minimum in the range of φ_2_ = 30° to 0° was observed with Δ*E*=3.66 kcal·mol^−1^ (Δ*G* = 3.28 kcal·mol^−1^) higher than that of the φ_2_ = 180.0° conformer. The δ_calc_ (^1^Η) values of the low-energy conformers with φ_2_ = 30° to 0° and φ_2_ = 180.0° (Table S9), weighted by the respective Boltzmann factors, are essentially the same as those of the φ_2_ = 180.0° conformer. This is due to the negligible population of the higher minimum conformers. Of particular interest is the parallel behavior of the H_4_ and H_1b_ protons as a function of the torsion angle φ_2_, with strong deshielding in the range of 0° < φ_2_ < 90°. Again, the effect of the variation in the φ_2_ torsion angle on δ_calc_(^1^H), using the same level of theory as geometry optimization (APFD/6–311++G(d,p)), is to decrease slightly the Δ*Ε* and Δ*G* values and increase the deshielding effect (Figure S6 and Table S10).

**Figure 6:**
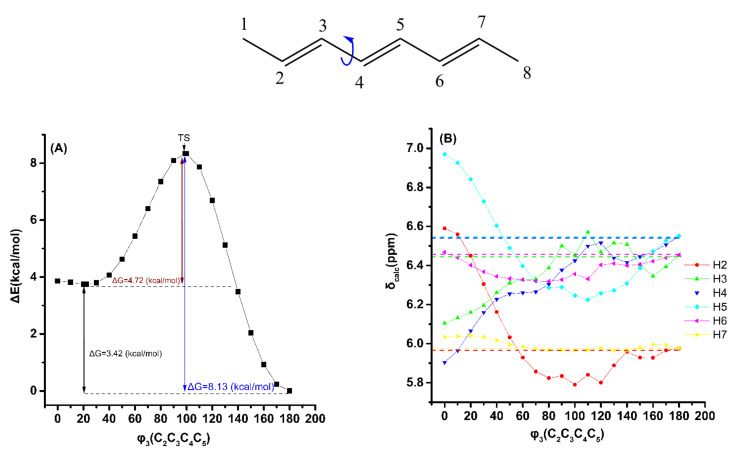
Effect of variation in the torsion angle φ_3_ (C_2_C_3_C_4_C_5_) of (*E*,*E,E*)-2,4,6-octatriene, with energy minimization at the B3LYP/6–31+G(d) level, on the electronic energy Δ*E* (kcal/mol) (characteristic Δ*G* values are also shown) (**A**) and on δ_calc_(^1^H) data of the olefinic protons (at the GIAO/B3LYP/6–311+G(2d,p) level of theory with CPCM in CCl_4_) (**B**). The experimental chemical shift values are denoted with the horizontal dotted lines.

Figure 6 shows the effect of variation in the torsion angle φ_3_(C_2_C_3_C_4_C_5_) of the (*E*,*E,E*)-2,4,6-octatriene, with energy minimization at the B3LYP/6–31+G(d) level, on the electronic energy Δ*Ε*, and the computational olefinic ^1^H NMR chemical shifts (at the GIAO/B3LYP/6–311+G(2d,p) level with CPCM in CHCl_3_). As in the case of (*E*)-1,3,5-hexatriene, a second broad minimum in the range of φ_3_ = 30° to 0° was observed with Δ*E* = 3.68 kcal·mol^−1^ (Δ*G* = 3.42 kcal·mol^−1^). The δ_exp_(^1^H) data of the olefinic protons (Table S1) are in excellent agreement with the δ_calc_(^1^H) values of the minimum energy conformer with φ_3_ = 180.0^o^. The δ_calc_(^1^H) values of the low-energy conformers (Table S11), weighted by the respective Boltzmann factor, are essentially the same as those of the φ_3_ = 180.0° conformer. This demonstrates that the weights of the higher minimum conformers are of minor importance. As in the previous cases, the effect of variation in the φ_3_ torsion angle on δ_calc_(^1^H) of the olefinic protons, using the same level of theory as geometry optimization (APFD/6–311++G(d,p)), is to decrease slightly the Δ*Ε* and Δ*G* values and increase the deshielding effect (Figure S7 and Table S12).

It can, therefore, be concluded that δ_exp_(^1^H) are reproduced very accurately for the lowest-energy DFT-optimized single conformer. The other low-energy conformers have negligible effects on the δ_calc_(^1^H) data of the conjugated olefinic protons. Furthermore, the minor functional dependence can result in the levels of accuracy that are necessary for structure elucidation in solution (see below).

Abraham et al. [50] showed that there is a deshielding effect above the C=C bond at short distances, due to the van der Waals term, and a deshielding effect in the plane of the C=C bond [57]. Figure S8 illustrates a plot of NBO bond order of the olefinic C-H bonds vs. δ_calc_(^1^H). The resulting very poor correlation (*R*^2^ = 0.554) shows that the NBO bond order is not a significant factor that determines δ_calc_(^1^H). Furthermore, no functional relationship was found for NBO charge densities of the olefinic C-H protons vs. δ_calc_(^1^H). Α detailed experimental and computational (with the CHARGE 7 model) study by Abraham et al. [50,57] showed a steric deshielding effect on both alkene and aromatic ring protons, contrary to a shielding effect of H^…^H steric interactions in alkanes. All these steric interactions were described by a simple *r*^−6^ dependence,

δ_steric_ = *a*_s_/*r*^6^

where *a*_s_ is a constant of the given hybridization of the atom. Figure S9 and Figure S10 show that, in most cases, an *r^-n^* dependence is observed, which for H^…^H distances shorter than those of the van der Waals radius of the hydrogen atom (~1.2 Å), becomes approximately a linear correlation. According to the semi empirical model of Cheney [51], a much better agreement with the experimental deshielding effect was obtained by correlation with *f*(*r_i_*, *θ_i_*) than with the exponential exp(−*ar_i_*) term, where *r_i_* is the distance between the interacting pair of hydrogens and *θ_i_* is the angle between H^…^H internuclear line and the H–C bond of interest. The above results clearly demonstrate that the deshielding effect due to non-bonded H^…^H repulsive interaction is a primary factor affecting δ(^1^H) in the trienyl systems reported therein, as in the case of simple alkenes, conjugated dienyl, and aromatic systems [47,50,51,57].

### 2.3. DFT-Calculated vs. Experimental ^1^H NMR Chemical Shifts: Structure Elucidation in Solution of Geometric Isomers of 18:3 Conjugated Linolenic Acids and Hexadecatrienyl Pheromones

**Figure 7:**
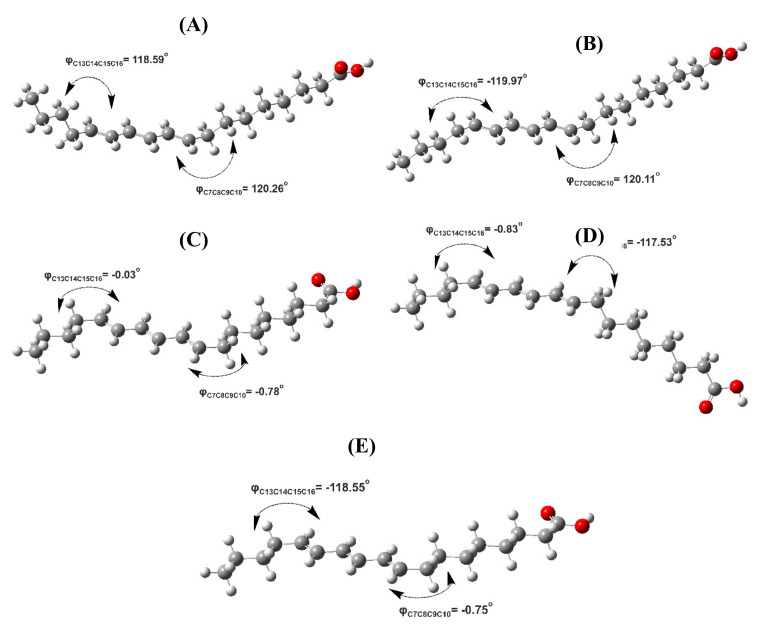
Structures of various conformers (**A**–**E**) of the β-eleostearic acid (9*E*,11*E*,13*E*-isomer) with energy minimization in the gas phase at the B3LYP/6–31+G(d) level of theory. Δ*G* values (kcal·mol^−1^) and % populations of conformers (**A**–**E**) are shown in Table 2.

**Figure 8:**
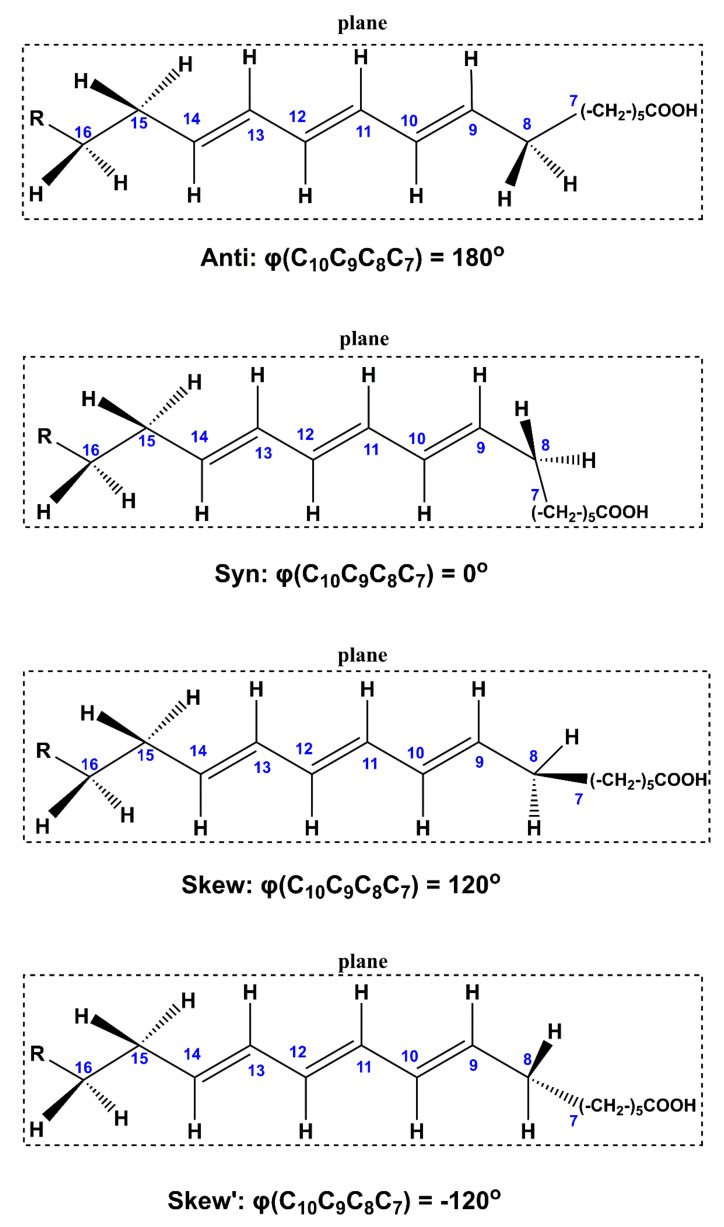
Definition of various conformations of the allylic carbon C8 of β-eleostearic acid (9*E*,11*E*,13*E* isomer).

Table 2 shows conformational properties, Δ*G* values (kcal·mol^−1^) and % populations of various low-energy conformers of the 18:3 ω-5 CLA with energy minimization at the B3LYP/6–31+G(d) and APFD/6–31+G(d) levels. The structures of five low-energy conformers (A), (B), (C), (D), and (E) of β-eleostearic acid and the definition of various torsion angles are shown in Figure 7 and Figure 8, respectively. The general tendency of the torsion angles φ(C_7_C_8_C_9_C_10_) and φ(C_13_C_14_C_15_C_16_), which involve the allylic C(8) and C(14) carbons, respectively, is to adopt a low-energy gauche conformation, also known as skew (*S* = 120°) or skew′ (*S*′ = −120°). On the contrary, the eclipsed syn conformation (φ = 0°) results in high energy and, thus, low population (Table 2). The results are very similar for both B3LYP/6–31+G(d) and APFD/6–31+G(d) levels. An anti-zigzag conformational behavior was observed for the two polymethylene (CH_2_)_n_ groups, which are attached to the conjugated trienyl system, for all conformers of the geometric isomers of 18:3 ω-5 CLA for both B3LYP/6–31+G(d) and APFD/6–31+G(d) levels (Table 2 and Figure S11).

Crystallographic data for *cis*-monounsaturated fatty acids [58,59,60] revealed that the zigzag (anti) conformation of the polymethylene chains (CH_2_)_n_ is the most rigid and stable, whereas the gauche conformer is less stable, in agreement with our computational data. For the allylic carbons, the most stable conformations are skew (*S*) and skew′ (*S*′) in the γ-crystallization form, again in agreement with our computational data of Table 2. However, the anti-*cis*-anti and skew-*cis*-anti conformations have been observed in the α- and β-crystallization forms, respectively [58]. The formation of these high-energy conformers can be attributed to specific crystal packing interactions, which are absent in solution.

Figure S12 shows a graphical presentation of the calculated ^1^H NMR chemical shifts (at the GIAO/B3LYP/6–311+G(2d,p) (CPCM, CHCl_3_) level), weighted by the respective Boltzmann factors of the various conformers of Table 2 vs. experimental chemical shifts (Table 1 and Table S13) of the three geometric isomers of the 9,11,13-CLA. The experimental chemical shifts of the H11 and H12 of β-eleostearic acid [18] deviate from linearity. Revision of the literature assignment so that H12 is deshielded to a larger degree than H11 results in very good agreement with the computational data and significant improvement of the statistical data (Table S13 and Table S14). Similar results were obtained with calculations of ^1^H NMR chemical shifts using the same level of theory as geometry optimization (APFD/6–31+G(d) level) (Table S14).

Table 2 shows conformational properties, Δ*G* values (kcal·mol^−1^), and % populations of various low-energy conformers of the 10,12,14-conjugated hexadecatrienyl acetate geometric isomers with geometry optimization at the B3LYP/6–31+G(d) and APFD/6–31+G(d) levels. As in the case of CLnAs, the torsion angle φ(C_7_C_8_C_9_C_10_) adopts a low-energy skew (120°) or skew′ (120°) conformation. On the contrary, the eclipsed syn conformation with φ angles around 0^o^ results in high energy and, thus, low population. Figure S13 and Table S15 and Table S16 show an improvement in the correlation of δ_calc_ vs. δ_exp_ when the literature experimental chemical shifts of H13 and H15 of 9*Z*,11*Z*,13*E* and H13 and H14 of 9*Z*,11*E*,13*E*-conjugated hexadecatrienyl acetates (Table 1, [13]) have been revised (Figure S14). Similar results were obtained with the calculation of ^1^H NMR chemical shifts using the same level of theory as geometry optimization (APFD/6–31+G(d) level) (Table S15 and Table S16).

**Figure 9:**
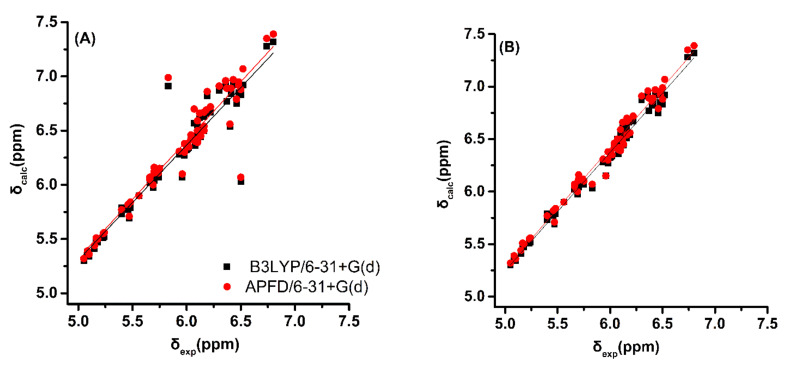
(**A**) Calculated, δ_calc_, ^1^H NMR chemical shifts of the olefinic protons at the GIAO/B3LYP/6–311+G(2d,p) level with CPCM) vs. experimental, δ_exp_, chemical shifts with energy minimization using B3LYP/6–31+G(d) and APFD/6–31+G(d) for *(Z*)-1,3,5-hexatriene, (*E*)-1,3,5-hexatriene, (*E,Z,E*)-2,4,6-octatriene, (*E,E,E*)-2,4,6-octatriene, α-oleostearic acid (9*Z*, 11*E*, 13*E*), *β*-oleostearic acid (9*E*, 11*E*, 13*E*), punicic acid (9*Z*, 11*E*, 13*Z*), and (10*E*, 12*E*, 14*Z*)-, (10*E*,12*Z*,14*Z*)-, (10*Z*, 12*Z*, 14*E*)-, and (10*Z*, 12*E*, 14*E*)-hexatrienyl acetates. (**B**) The same as in (**A**); however, the literature experimental chemical shift data (δ_exp_) of the compounds shown in Figure S14 have been revised.

Figure 9 and Table 3 show that a significant improvement can be achieved in the linear regression correlation coefficient and mean square error of the correlation δ_calc_ vs. δ_exp_ of the olefinic protons of all the compounds of Figure 1 when the literature experimental chemical shifts of the compounds shown in Figure S14 have been revised. This clearly demonstrates that the accuracy of the DFT ^1^H NMR shift prediction can be used to resolve ambiguities in resonance assignment.

## 3. Computational Methods

The computational study was performed using the Gaussian 09 Rev. D.01 software [61]. The initial structures of the model triethyl compounds (*Z*)-1,3,5-hexatriene, (*E*)-1,3,5-hexatriene, (*E,Z,E*)-2,4,6-octatriene, and (*E,E,E*)-2,4,6-octatriene were drawn using the GaussView program and were energy minimized at the B3LYP/6–31+G(d), B3LYP/6–311++G(d,p), B3LYP-D3/6–31+G(d), B3LYP-D3/6–311++G(d,p) (the D3 version of Grimme dispersion with Becke–Johnson damping), APFD/6–31+G(d), APFD/6–311++G(d,p), PBE0/6–31+G(d), PBE0/6–311++G(d,p), M06–2X/6–31+G(d), M06–2X/6–311++G(d,p), *ω*B97XD/6–31+G(d), and *ω*B97XD/6–311++G(d,p) levels (gas phase). In the case of α-oleostearic acid (9*Z*, 11*E*, 13*E*), *β*-oleostearic acid (9*E*, 11*E*, 13*E*), punicic acid (9*Z*, 11*E*, 13*Z*), (10*E*, 12*E*, 14*Z*)-, (10*E*,12*Z*,14*Z*)-, (10*Z*, 12*Z*, 14*E*)-, and (10*Z*, 12*E*, 14*E*)-hexatrienyl acetates, a conformational analysis was, firstly, performed by rotation of the aliphatic carbons of the (CH_2_)_n_ chains, which constitute with the rigid conjugated double bonds a flat system. The resulting conformers were energy minimized with DFT at the B3LYP/6–31+G(d) and APFD/6–31+G(d) levels, while the subsequent energy minimization of the selected stable structures was carried out at the same level of calculation. The optimized geometries were verified by performing frequency calculation at the same level (zero imaginary frequencies). The scanning of torsional angles was performed using the redundant coordinates in Gaussian 09 [47]. Δ*G* values were calculated from the thermochemistry results, either between the stable conformers or between the stable conformers and the corresponding transition states. The computed proton chemical shifts were performed using (i) a single methodology at the GIAO/B3LYP/6–311+G(2d,p) level and (ii) GIAO at the same level of theory as geometry optimization. In both cases, the conductor-like polarizable continuum model (CPCM) was used in CHCl_3_ or CCl_4_, for experimental values obtained in CDCl_3_ and CCl_4_, respectively (Table S1 and Table S2). δ_calc_(^1^Η) were referenced with respect to the standard TMS, which was optimized at the same level (Table S17).

## 4. Conclusions

From the DFT data of the trienyl conjugated compounds of Figure 1 reported therein, it can be concluded that:

(a) Very good linear correlations can be obtained between DFT-calculated and experimental ^1^H NMR chemical shifts of the olefinic protons of the lowest-energy DFT-optimized single conformer using standard functionals (B3LYP and PBE0) as well as corrections for dispersion interactions (B3LYP-D3, APFD, M06–2X and ωB97XD). The ωB97XD performs slightly better, but again the accuracy of the functionals used was rather similar.
(b) Through-space H^…^H steric interaction is the primary factor that results in strong deshielding of closely spaced trienyl olefinic protons, in excellent agreement with literature data on alkenes and aromatic systems [50,51,57].
(c) The accuracy of computational ^1^H NMR chemical shifts can facilitate (i) the unequivocal assignment of the geometric isomerism in conjugated trienyl systems of biological systems, such as CLnAs and hexadecatrienyl pheromones, and especially in the case of problematic resonance assignment due to extensive signal overlap, and (ii) structure elucidation in solution [28,36].

## Figures and Tables

**Table 1:** Literature experimental ^1^H NMR chemical shifts, δ_exp_, and the sample and spectral specifications of the compounds of Figure 1.

A		B	C	D	E		F	G		H	I	J	K		L	M
Group	δ_exp_ (ppm)	δ_exp_ (ppm)	δ_exp_ (ppm)	δ_exp_ (ppm)	Group	δ_exp_ (ppm)	δ_exp_ (ppm)	Group	δ_exp_ (ppm)	δ_exp_ (ppm)	δ_exp_ (ppm)	δ_exp_ (ppm)	Group	δ_exp_ (ppm)	δ_exp_ (ppm)	δ_exp_ (ppm)
**H1a**	5.15	5.1	5.09	5.05	**H1**	1.8	1.84	**H9**	2.09	2.12	2.18	2.17	**H11**	6.10	6.19	6.48
**H1b**	5.24	5.23	5.17	5.18	**H2**	5.7	5.69	**H10**	5.70	5.74	5.48	5.40	**H12**	6.10	6.40	6.48
**H2**	6.8	6.36	6.74	6.3	**H3**	5.83	6.12	**H11**	6.10	6.50	6.43	5.98	**H10**	6.04	6.01	6.08
**H3**	6.0	6.22	5.93	6.16	**H4**	6.5	6.10	**H12**	6.18	5.98	6.13	6.37	**H13**	6.04	6.12	6.08
**H4**	6.0	6.22	5.93	6.16	**H5**	6.5	6.10	**H13**	6.41	6.16	5.96	6.16	**H9**	5.66	5.4	5.46
**H5**	6.8	6.36	6.74	6.3	**H6**	5.83	6.12	**H14**	6.02	6.46	6.52	6.07	**H14**	5.66	5.74	5.46
**H6a**	5.15	5.1	5.09	5.05	**H7**	5.7	5.69	**H15**	5.47	5.56	5.75	5.70	**H2**	2.37	2.40	2.37
**H6b**	5.24	5.23	5.17	5.18	**H8**	1.8	1.84	**H16**	1.76	1.77	1.80	1.77	**H8**	2.10	2.20	2.22
													**H15**	2.10	2.20	2.22
													**H3**	1.65	1.64	1.65
													**H4**	1.39	1.41	1.39
													**H5**	1.39	1.41	1.39
													**H6**	1.39	1.41	1.39
													**H7**	1.39	1.41	1.39
													**H16**	1.33	1.33	1.32
													**H17**	1.33	1.33	1.32
													**H18**	0.91	0.97	0.94

^A^ (*Z*)-1,3,5-hexatriene (CDCl_3_), ^1^H and 2D ^1^H–^1^H COSY NMR (270 MHz) [15]; ^B^ (*E*)-1,3,5-hexatriene (CDCl_3_), ^1^H and 2D ^1^H–^1^H COSY NMR (270 MHz) [15]; ^C^ (*Z*)-1,3,5-hexatriene (CCl_4_), ^1^H NMR (100 MHz) [21,22]; ^D^ (*E*)-1,3,5-hexatriene (CCl_4_), ^1^H NMR (100 MHz) [21,22]; ^E^ (*E,Z,E*)-2,4,6-octatriene (CDCl_3_), ^1^H NMR (300 MHz) [23]; ^F^ (*E,E,E*)-2,4,6-octatriene (CCl_4_), ^1^H NMR (100 MHz) [21]; ^G^ 10*E*,12*E*,14*Z*-hexadecatrienyl acetate (CDCl_3_), ^1^H, ^13^C, and 2D ^1^H–^1^H COSY NMR (270 MHz) [13]; ^H^ 10*E*,12*Z*,14*Z*-hexadecatrienyl acetate (CDCl_3_), ^1^H, ^13^C, and 2D ^1^H–^1^H COSY NMR (270 MHz) [13]; ^I^ 10*Z*,12*Z*,14*E*-hexadecatrienyl acetate (CDCl_3_), ^1^H, ^13^C, and 2D ^1^H–^1^H COSY NMR (270 MHz) [13]; ^J^ 10*Z*,12*E*,14*E*-hexadecatrienyl acetate (CDCl_3_), ^1^H, ^13^C, and 2D ^1^H–^1^H COSY NMR (270 MHz) [13]; ^K^ β-eleostearic acid (CDCl_3_), ^1^H, ^13^C, and 2D ^1^H–^1^H COSY NMR (400 MHz) [18]; ^L^ α-eleostearic acid (CDCl_3_), ^1^H, ^13^C, and 2D ^1^H-^1^H COSY NMR (400 MHz) [18]; ^M^ punicic acid (CDCl_3_), ^1^H, ^13^C, and 2D ^1^H–^1^H COSY NMR (400 MHz) [18].

**Table 2:** Conformational properties, Δ*G* values (kcal·mol^−1^), and % populations of various low-energy conformers of the 9,11,13-conjugated fatty acid and 10,12,14-conjugated hexadecatrienyl acetate geometric isomers with geometry optimization at the B3LYP/6–31+G(d) and APFD/6–31+G(d) levels.

Compound	Conformer	B3LYP/6–31+G(d)			APFD/6–31+G(d)		
		φ_c7c8c9c10_ (°)	φ_c13c14c15c16_ (°)	Δ*G* (kcal/mol) (% Population)	φ_c7c8c9c10_ (°)	φ_c13c14c15c16_ (°)	Δ*G* (kcal/mol) (% Population)
9*E*,11*E*,13*E* Isomer (β-Eleostearic acid)	A	120.26 (S)	118.59 (S)	+0.36 (33.41)	119.21 (S)	117.99 (S)	+0.16 (38.68)
	B	120.11 (S)	−119.97 (S’)	0.00 (61.35)	118.73 (S)	−118.59 (S’)	0.00 (50.67)
	C	−0.78 (Syn)	−0.03 (Syn)	+3.00 (0.39)	−0.59 (Syn)	−0.11 (Syn)	+2.13 (1.39)
	D	−117.53 (S’)	−0.83 (Syn)	+1.89 (2.53)	−116.94 (S’)	−0.62 (Syn)	+1.38 (4.94)
	E	−0.75 (Syn)	−118.55 (S’)	+1.94 (2.32)	−0.58 (Syn)	−117.27 (S’)	+1.46 (4.31)
9*Z*,11*E*,13*E* Isomer (β-Eleostearic acid)	A	116.67 (S)	119.18 (S)	+0.03 (25.07)	111.25 (S)	117.87 (S)	0.00 (36.53)
	B	−118.92 (S’)	−119.37 (S’)	+0.15 (20.48)	−114.31 (S’)	−117.53 (S’)	+0.48 (16.25)
	C	116.88 (S)	−119.16 (S’)	+0.02 (25.50)	112.96 (S)	−117.54 (S’)	+0.18 (26.96)
	D	−118.88 (S’)	119.28 (S)	0.00 (26.38)	−113.83 (S’)	118.32 (S)	+0.47 (16.52)
	E	−119.43 (S’)	−0.76 (Syn)	+1.38 (2.57)	−113.80 (S’)	−0.22 (Syn)	+1.35 (3.74)
9Z,11*E*,13Z Isomer (Punicic acid)	A	117.46 (S)	119.33 (S)	0.00 (38.54)	113.45 (S)	114.70 (S)	0.00 (44.84)
	B	117.76 (S)	−118.63 (S’)	+0.08 (33.67)	112.18 (S)	−112.33 (S’)	+0.31 (26.57)
	C	−119.22 (S’)	119.55 (S)	+0.27 (24.43)	−113.43 (S’)	113.54 (S)	+0.27 (28.43)
	D	1.52	−121.09 (S’)	+3.83 (1.51)	5.34	−110.17 (S’)	+3.77 (0.08)
	E	117.81 (S)	2.02 (Syn)	+3.63 (1.85)	111.01 (S)	4.55 (Syn)	+3.78 (0.08)
10*E*,12*E*,14*Z*-Hexadecatrienyl acetate	A	120.04 (S)		+0.28 (25.59)	118.73 (S)		+0.08 (28.88)
	B	−119.39 (S’)		0.00 (41.05)	−118.73 (S’)		+0.03 (31.42)
	C	120.03 (S)		+0.20 (29.29)	118.73 (S)		0.00 (33.05)
	D	1.56 (Syn)		+1.37 (4.07)	1.16 (Syn)		+0.95 (6.65)
10*E*,12Z,14Z-Hexadecatrienyl acetate	A	119.67 (S)		+0.44 (22.44)	118.45 (S)		+0.20 (26.06)
	B	−119.64 (S’)		0.00 (47.15)	−118.86 (S’)		0.00 (36.52)
	C	119.56 (S)		+0.35 (26.12)	118.28 (S)		+0.12 (29.82)
	D	1.22 (Syn)		+1.42 (4.29)	0.87 (Syn)		+0.93 (7.60)
10Z,12Z,14*E*-Hexadecatrienyl acetate	A	119.01 (S)		0.00 (30.52)	112.30 (S)		+0.04 (20.48)
	B	−119.51 (S’)		+0.51 (12.91)	−113.47 (S’)		+0.10 (18.51)
	C	118.80 (S)		0.00 (30.52)	112.24 (S)		0.00 (21.92)
	D	−119.50 (S’)		+0.48 (13.57)	−113.16 (S’)		+0.02 (21.19)
10Z,12*E*,14*E*-Hexadecatrienyl acetate	A	119.05 (S)		0.00 (31.98)	113.03 (S)		0.00 (33.79)
	B	−121.15 (S’)		+0.54 (12.85)	−116.62 (S’)		+0.63 (11.67)
	C	118.73 (S)		+0.04 (29.89)	113.00 (S)		+0.04 (31.59)
	D	−121.06 (S’)		+0.56 (12.43)	−116.57 (S’)		+0.65 (11.28)

**Table 3:** Linear regression correlation coefficient, mean square error, intercept, and slope of the calculated vs. experimental olefinic ^1^H NMR chemical shifts of Figure 9.

Minimization Method	Correlation Coefficient (*R*^2^)	Mean Square Error	Intercept	Slope
**B3LYP/6–31+G(d) ^a^**	0.873	0.037	−0.002	1.062
**APFD/6–31+G(d) ^a^**	0.868 (0.825)	0.041(0.044)	−0.102 (0.518)	1.085 (0.956)
**B3LYP/6–31+G(d) ^b^**	0.984	0.005	−0.387	1.127
**APFD/6–31+G(d) ^b^**	0.982 (0.942)	0.005 (0.015)	−0.506 (0.138)	1.153 (1.020)

^a^ Data of Figure 9A; calculation of ^1^H NMR chemical shifts at the GIAO/B3LYP/6–311+G(2d,p) level and ^b^ data of Figure 9B; calculation of ^1^H NMR chemical shifts at the GIAO/B3LYP/6–311+G(2d,p) level. The data in parenthesis were obtained at the GIAO with the same level of theory as geometry optimization.

## Supplementary Materials

Figure S1: Calculated ^1^H NMR chemical shifts vs. experimental chemical shifts, Figure S2: Calculated ^1^H NMR chemical shifts vs. experimental chemical shifts, Figure S3: Calculated ^1^H NMR chemical shifts vs. experimental chemical shifts, Figure S4: Effect of variation of the torsion angle φ (C_1_C_2_C_3_C_4_), Figure S5: Effect of variation of the torsion angle φ_1_(C_2_C_3_C_4_C_5_), Figure S6: Effect of variation of the torsion angle φ_2_(C_1_C_2_C_3_C_4_), Figure S7: Effect of variation of the torsion angle φ_3_ (C_2_C_3_C_4_C_5_), Figure S8: ΝΒO bond order of the olefinic C-H bonds vs. calculated ^1^H NMR chemical shifts, Figure S9: The dependence of δ(^1^H) vs. distance, Figure S10: The dependence of δ(^1^H) vs. distance, Figure S11: Structures of various conformers of the punicic acid, Figure S12: Graphical presentation of calculated ^1^H NMR chemical shifts vs. experimental values, Figure S13 Graphical presentation of calculated ^1^H NMR chemical shifts vs. experimental values, Figure S14: Experimental ^1^H NMR and calculated chemical shifts, Table S1: Experimental and computational ^1^H NMR chemical shifts, Table S2: Experimental and computational ^1^H NMR chemical shifts, Table S3: Statistical data of calculated vs. experimental ^1^H chemical shifts, Table S4: Statistical data of calculated vs. experimental ^1^H chemical shifts, Table S5: Effect of variation of the torsion angle φ(_1_C_2_C_3_C_4_) on calculated ^1^H NMR chemical shifts, Table S6: Effect of variation of the torsion angle φ(C_1_C_2_C_3_C_4_) on calculated ^1^H NMR chemical shifts, Table S7: Effect of variation of the C_2_C_3_C_4_C_5_ torsion angle on calculated ^1^H NMR chemical shifts, Table S8: Effect of variation of the C_2_C_3_C_4_C_5_ torsion angle on calculated ^1^H NMR chemical shifts, Table S9: Effect of variation of the C_1_C_2_C_3_C_4_ torsion angle on calculated ^1^H NMR chemical shifts, Table S10: Effect of variation of the C_1_C_2_C_3_C_4_ torsion angle on calculated ^1^H NMR chemical shifts, Table S11: Effect of variation of the C_2_C_3_C_4_C_5_ torsion angle on calculated ^1^H NMR chemical shifts, Table S12: Effect of variation of the C_2_C_3_C_4_C_5_ torsion angle on calculated ^1^H NMR chemical shifts, Table S13: Calculated and experimental ^1^H NMR chemical shifts, Table S14: Statistical analysis of the chemical shift data, Table S15: Calculated and experimental ^1^H NMR chemical shifts, Table S16: Statistical analysis of the chemical shift data, Table S17: Calculated shielding values of the reference TMS molecule.

## Sample Availability

Samples of the compounds are not available from the authors.
