# Supplementary material for: DFT Calculations of 1H NMR Chemical Shifts of Geometric Isomers of Conjugated Linolenic Acids, Hexadecatrienyl Pheromones, and Model Triene-Containing Compounds: Structures in Solution and Revision of NMR Assignments
Source: Molecules. 2021 Jun 7;26(11):3477. doi: 10.3390/molecules26113477 (PMC8201138; doi:10.3390/molecules26113477)
Supplement: Supplementary file 1 [file molecules-26-03477-s001.zip › molecules-1246095-supplementary.pdf]

Supplementary Material

DFT Calculations of ^{1}H NMR Chemical Shifts of Geometric Isomers of Conjugated Linolenic Acids, Hexadecatrienyl Pheromones, and Model Triene-Containing Compounds: Structures in Solution and Revision of NMR Assignments

Themistoklis Venianakis, Christina Oikonomaki, Michael G. Siskos *, Alexandra Primikyri and Ioannis P. Gerothanassis *

Section of Organic Chemistry and Biochemistry, Department of Chemistry, University of Ioannina, GR-45110 Ioannina, Greece; t.venianakis@uoi.gr (T.V.); pch01328@uoi.gr (C.O.); a.primikyri@uoi.gr (A.P.)

* Correspondence: msiskos@uoi.gr (M.G.S.); igeroth@uoi.gr (I.P.G.)

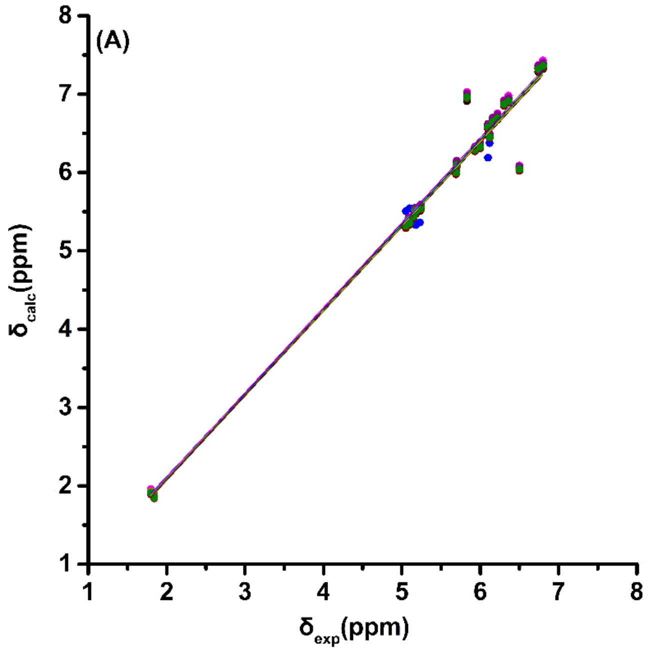

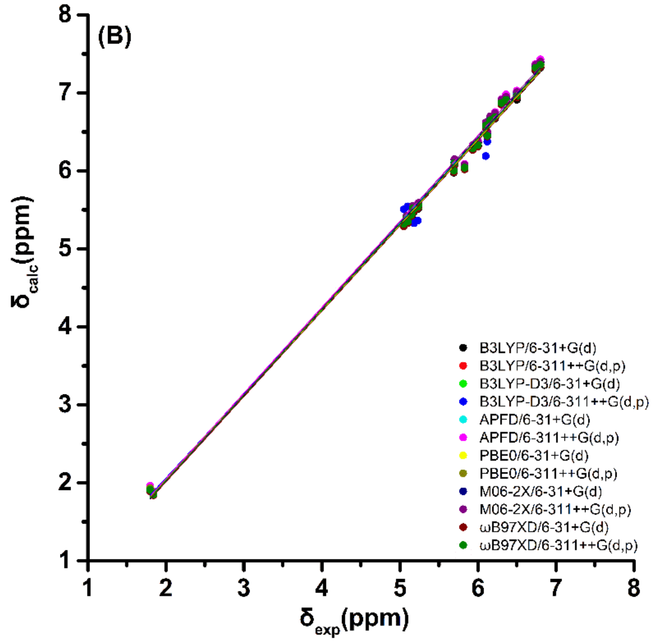

Figure S1. (A) Calculated, δ_{calc}, ^{1}H-NMR chemical shifts of the olefinic protons (at the GIAO/B3LYP/6-311+G(2d,p) level of theory with CPCM) *vs*. experimental, δ_{exp}, chemical shifts with energy minimization using various functionals and basis sets for the model trienyl compounds *(Z)*-1,3,5-hexatriene, (*E*)-1,3,5-hexatriene, (*E,Z,E*)-2,4,6-octatriene and (*E,E,E*)-2,4,6-octatriene. (**B**) As in (**A**), but the literature experimental values of δ(H(3,6)) = 5.83 ppm and δ(H(4,5)) = 6.50 ppm, of (*E,Z,E*)-2,4,6-octatriene [23] have been reversed.

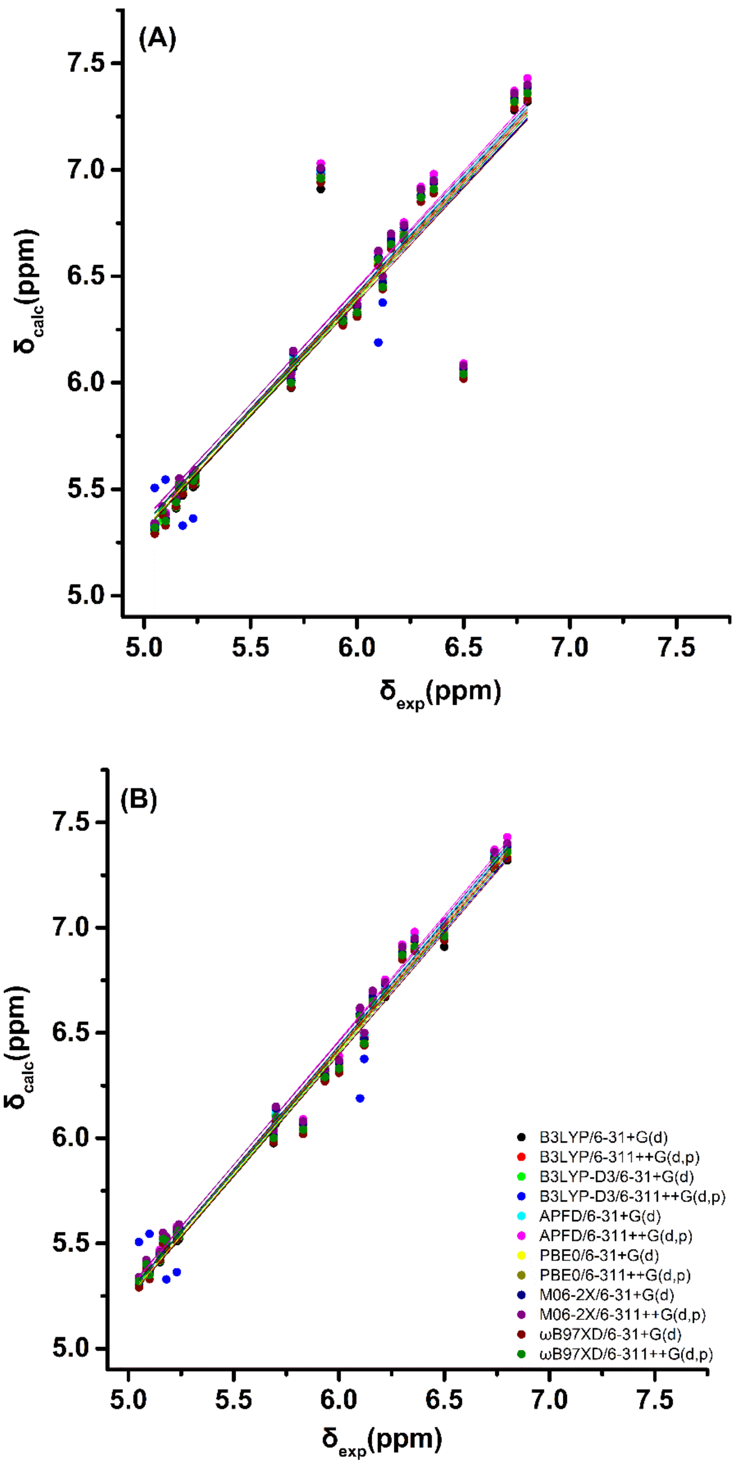

Figure S2. (**A**) Calculated, δ_{calc}, ^{1}H-NMR chemical shifts at the GIAO/B3LYP/6-311+G(2d,p) level of theory with CPCM) of the olefinic protons *vs*. experimental, δ_{exp}, chemical shifts with energy minimization using various functionals and basis sets for the model trienyl compounds *(Z)*-1,3,5-hexatriene, (*E*)-1,3,5-hexatriene, (*E,Z,E*)-2,4,6-octatriene and (*E,E,E*)-2,4,6-octatriene. (**B**) as in (A), but the literature experimental values of δ(H (3,6)) = 5.83 ppm and δ(H (4,5)) = 6.50 ppm, of (*E,Z,E*)-2,4,6-octatriene [23] have been reversed.

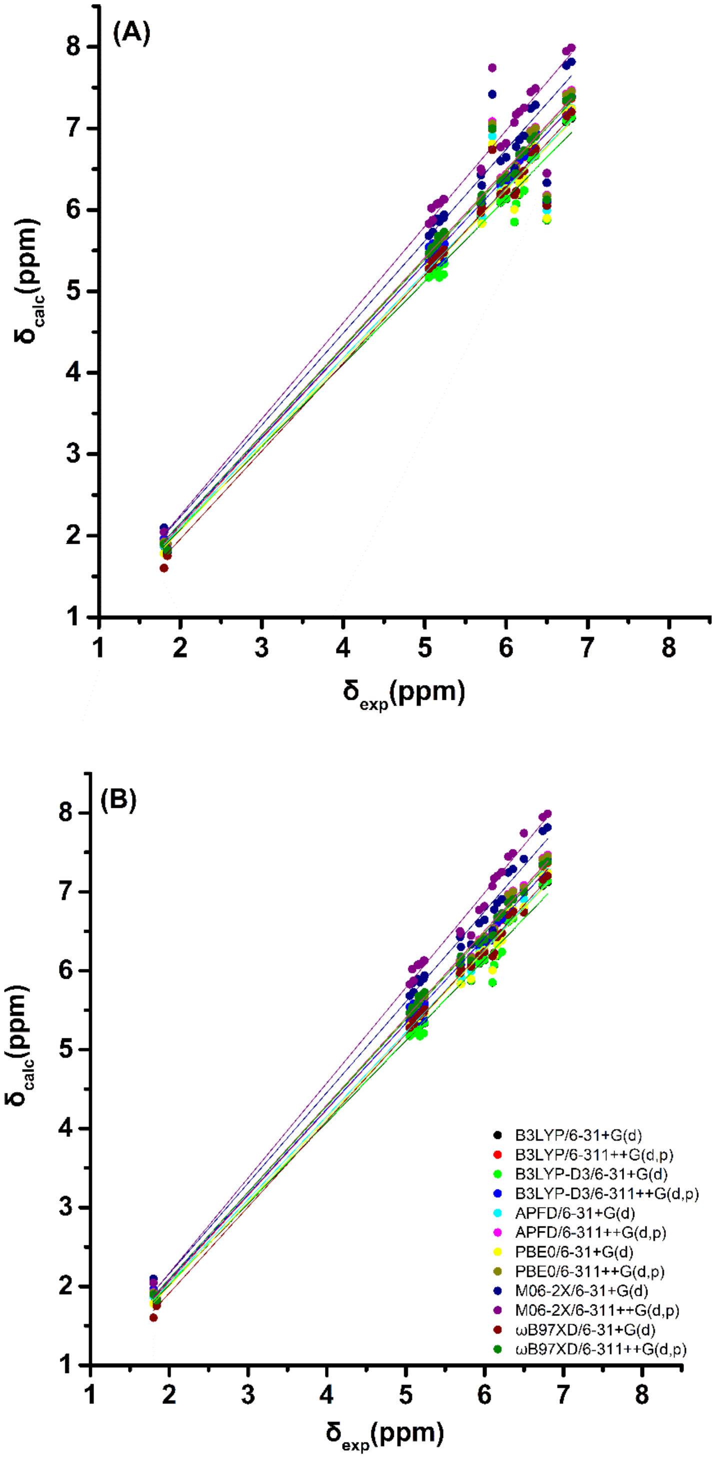

Figure S3. (A) Calculated, δ_{calc}, ^{1}H-NMR chemical shifts of the olefinic protons (with CPCM) *vs*. experimental, δ_{exp}, chemical shifts using the same level of theory as geometry optimization for the model trienyl compounds *(Z)*-1,3,5-hexatriene, (*E*)-1,3,5-hexatriene, (*E,Z,E*)-2,4,6-octatriene and (*E,E,E*)-2,4,6-octatriene. (**B**) As in (**A**), but the literature experimental values of δ(H(3,6)) = 5.83 ppm and δ(H(4,5)) = 6.50 ppm, of (*E,Z,E*)-2,4,6-octatriene [23] have been reversed.

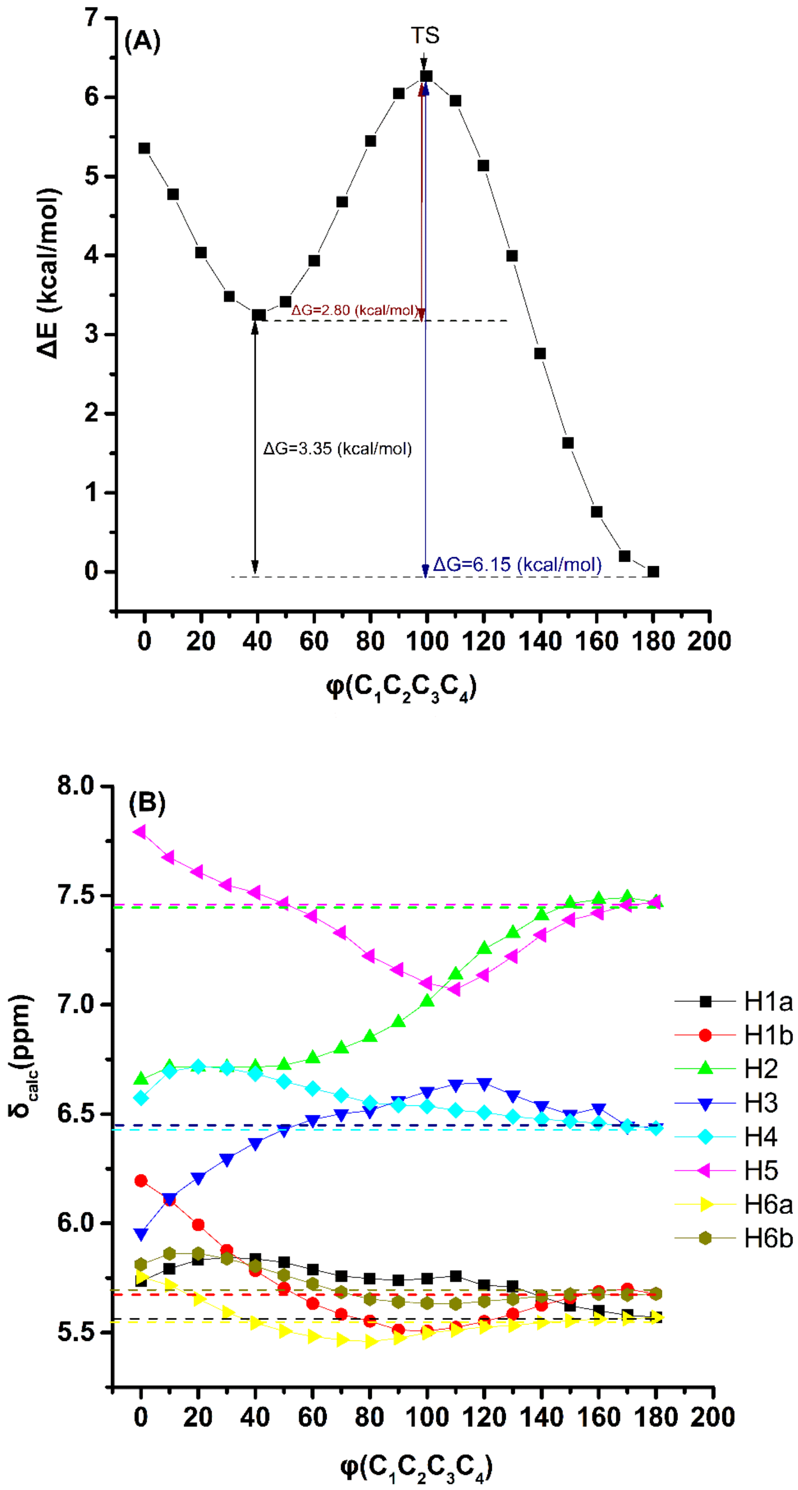

Figure S4. Effect of variation of the torsion angle φ ($C_1C_2C_3C_4$) of (*Z*)-1,3,5-hexatriene, on the electronic energy ΔE (kcal/mol) (characteristic ΔG values are also shown) (**A**), and computational ^{1}H-NMR chemical shifts with CPCM in $CHCl_3$ of the olefinic protons using the same level of theory as energy minimization (APFD/6-311++G(d,p)) (**B**). The experimental chemical shift values are denoted with the horizontal dotted lines.

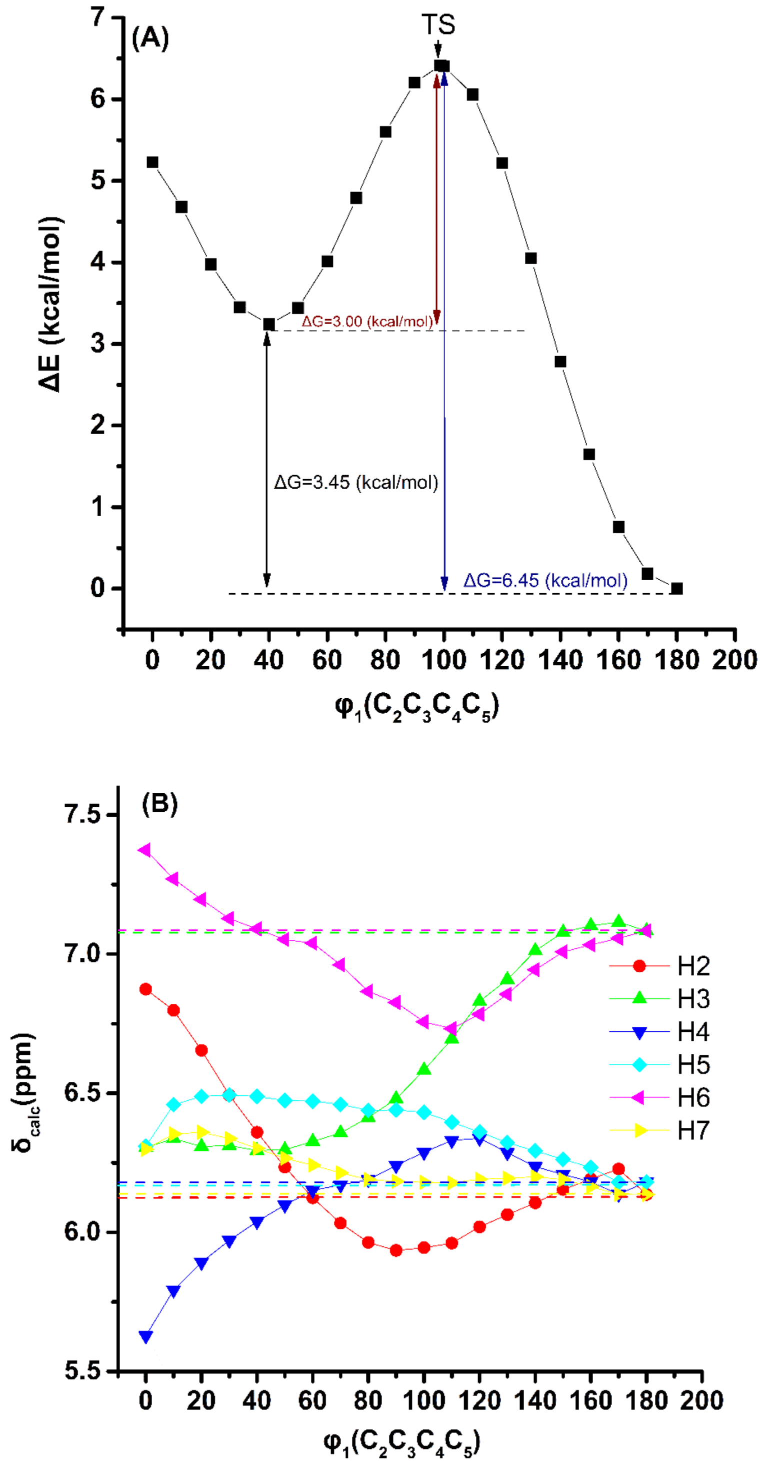

Figure S5. Effect of variation of the torsion angle $\varphi_1(C_2C_3C_4C_5)$ of (*E*,*Z*,*E*)-2,4,6-octatriene, on the electronic energy ΔE (kcal/mol) (characteristic ΔG values are also shown) (**A**) and computational olefinic ^{1}H-NMR chemical shifts with CPCM in $CHCl_3$ using the same level of theory as energy minimization (APFD/6-31++G(d,p)) (**B**). The experimental chemical shift values are denoted with the horizontal dotted lines.

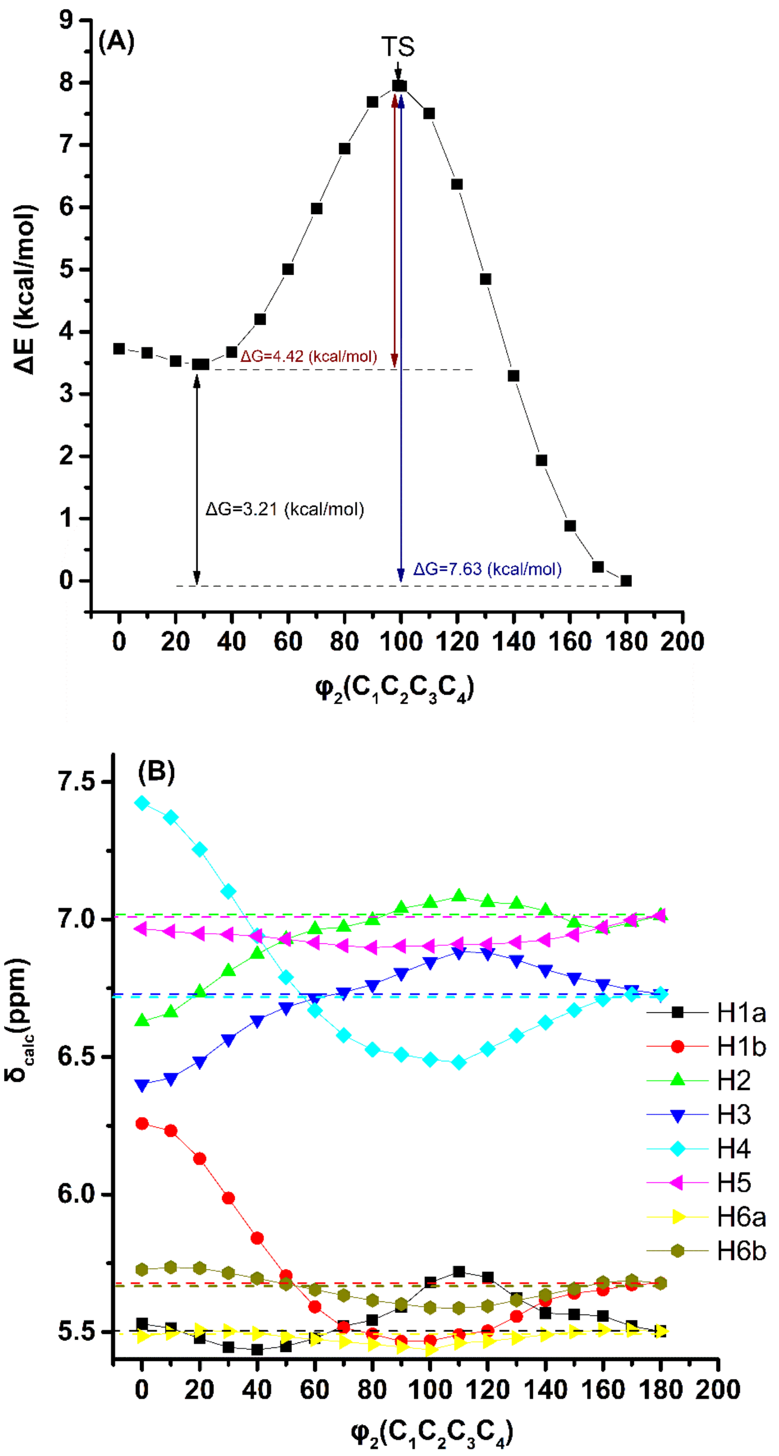

Figure S6. Effect of variation of the torsion angle $\varphi_2(C_1C_2C_3C_4)$ of (*E*)-1,3,5-hexatriene, on the electronic energy ΔE (kcal/mol) (characteristic ΔG values are also shown) (**A**) and computational olefinic ^{1}H-NMR chemical shifts with CPCM in $CHCl_3$ using the same level of theory as energy minimization (APFD/6-31++G(d,p)) (**B**). The experimental chemical shift values are denoted with the horizontal dotted lines.

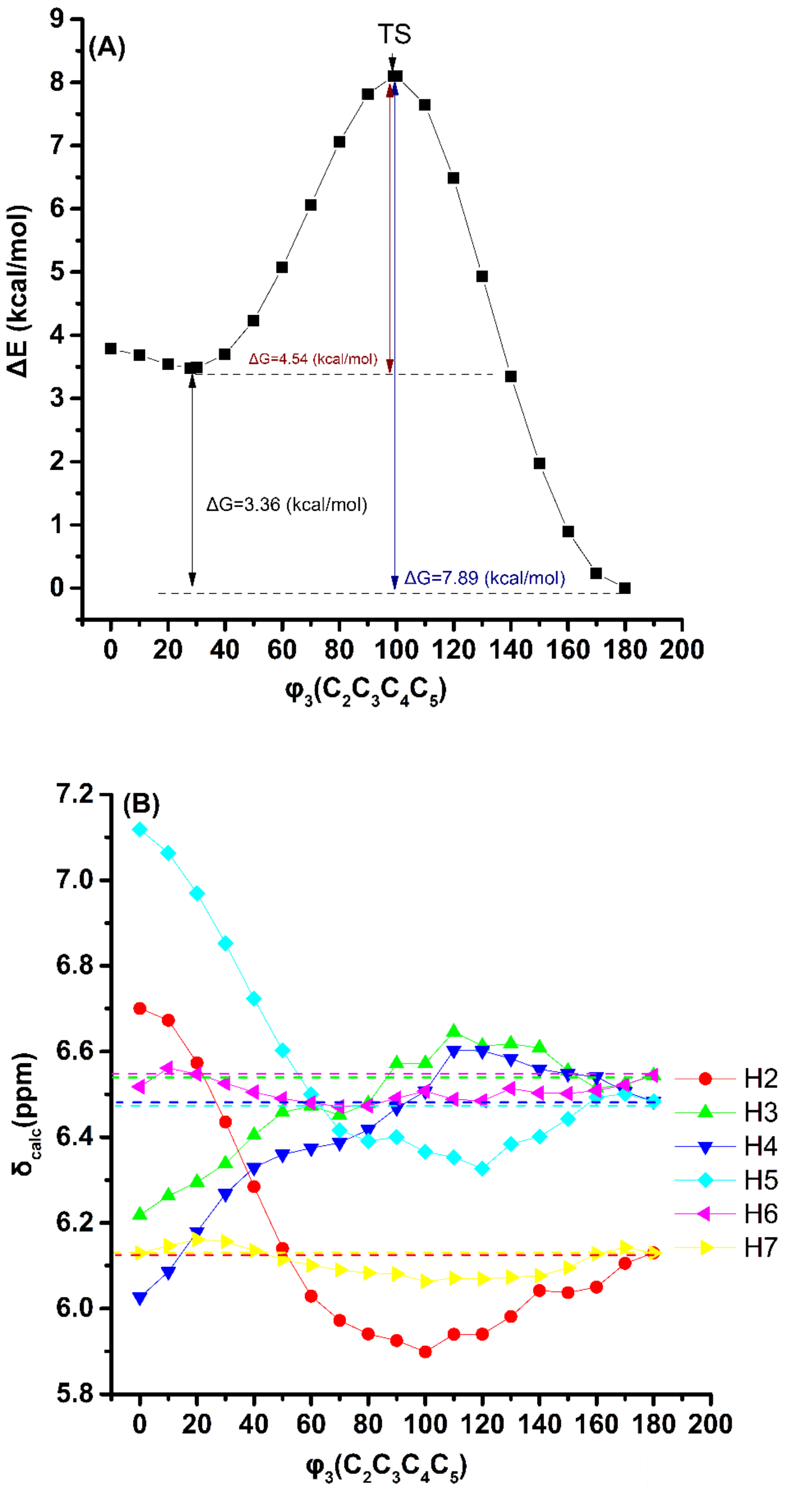

Figure S7. Effect of variation of the torsion angle φ_3 ($C_2C_3C_4C_5$) of (*E*,*E*,*E*)-2,4,6-octatriene, on the electronic energy ΔE (kcal/mol) (characteristic ΔG values are also shown) (**A**) and ^{1}H-NMR chemical shifts with CPCM in CCl_4 using the same level of theory as energy minimization (APFD/6-31++G(d,p) (**B**). The experimental chemical shift values are denoted with the horizontal dotted lines.

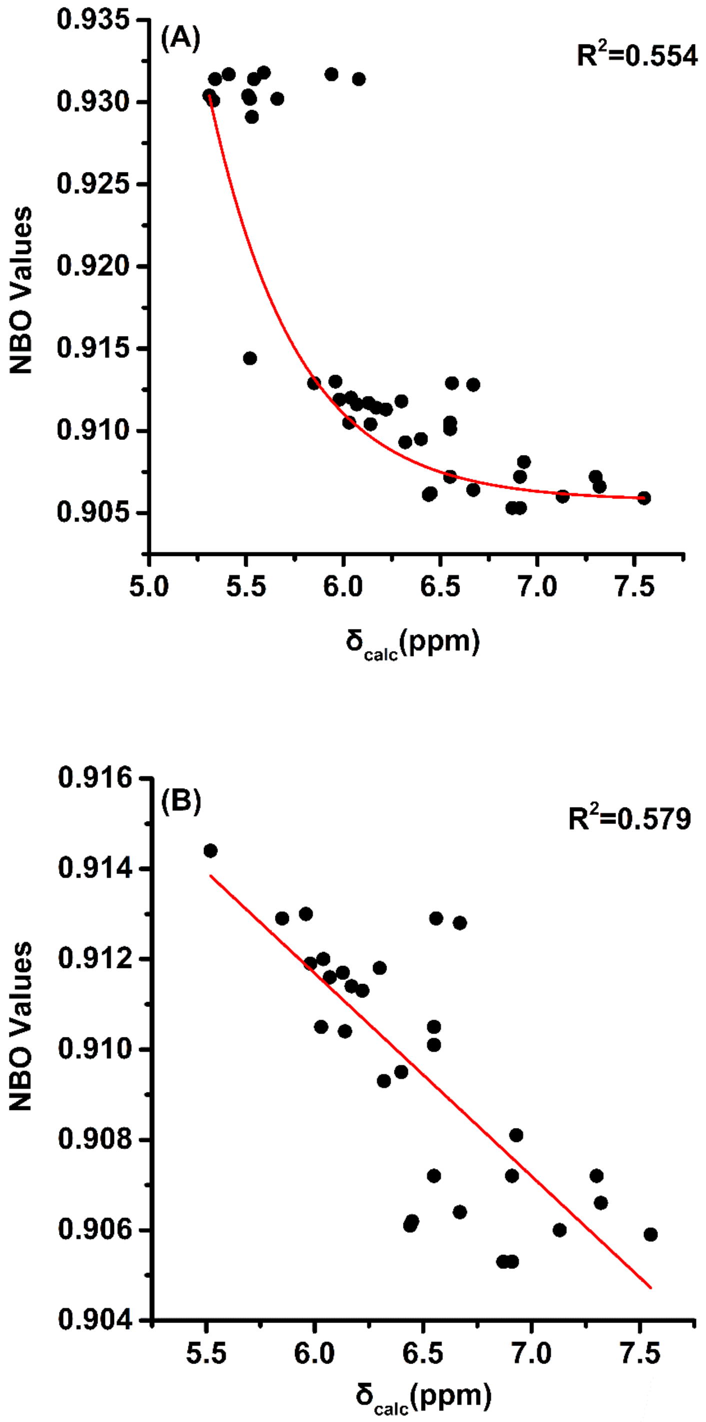

Figure S8. (**A**) NBO bond order of the olefinic C-H bonds of the model compounds (*Z*)-1,3,5- hexatriene, (*E*)-1,3,5- hexatriene, (*E,Z,E*)-2,4,6-octatriene, (*E,E,E*)-2,4,6-octatriene *vs*. calculated, δ_{calc}, ^{1}H-NMR chemical shifts at the B3LYP/6-31+G(d) level. (**B**) As in (**A**), however, the terminal CH_2 protons have been omitted.

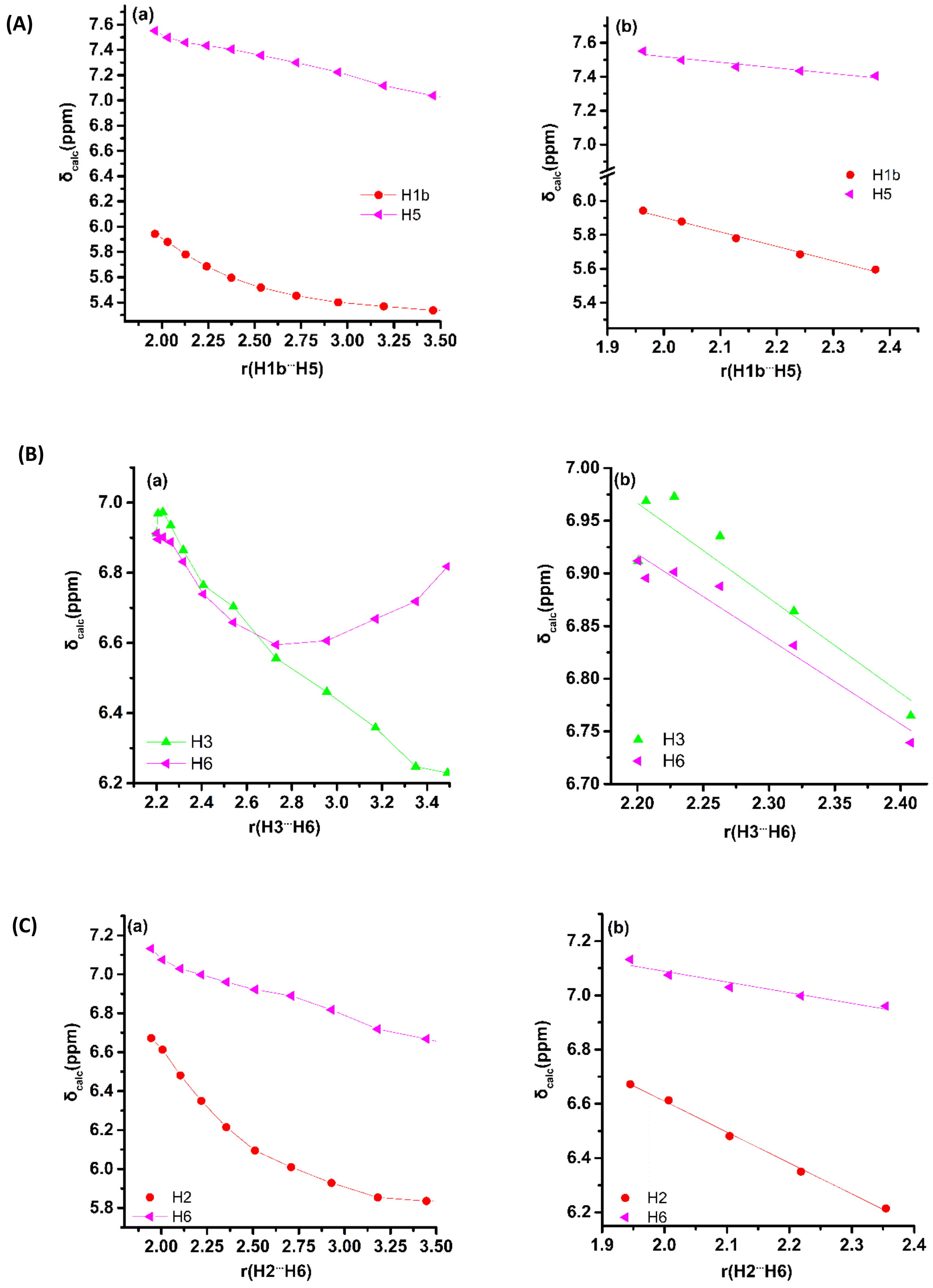

Figure S9. The dependence of δ(H1b, H5) *vs.* distance r(H1b···H5)of (*Z*)-1,3,5-hexatriene (**A**), δ(H3, H6) *vs.* r(H3···H6) of (*E,Z,E*)- 2,4,6- octatriene (**B**) and δ(H2, H6) *vs.* r(H2···H6) of (*E,Z,E*)-2,4,6- octatriene (**C**).

(A)

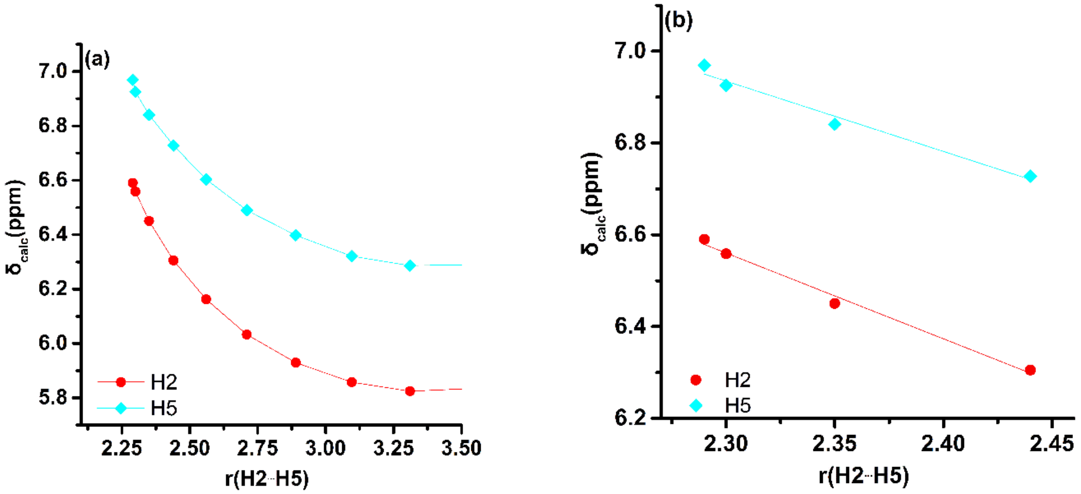

(B)

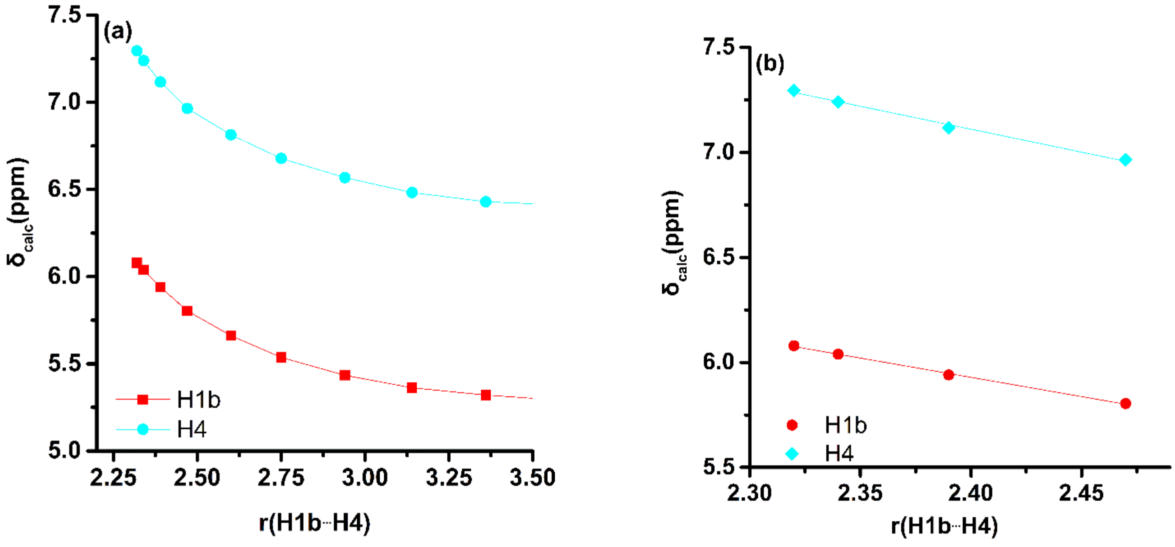

Figure S10. The dependence of δ(H2, H5) *vs.* distance r(H2···H5)of (*E,E,E*)- 2,4,6,- octatriene (**A**), and δ(H1b, H4) *vs.* r(H1b···H4) of (*E*)- 1,3,5- hexatriene (**B**).

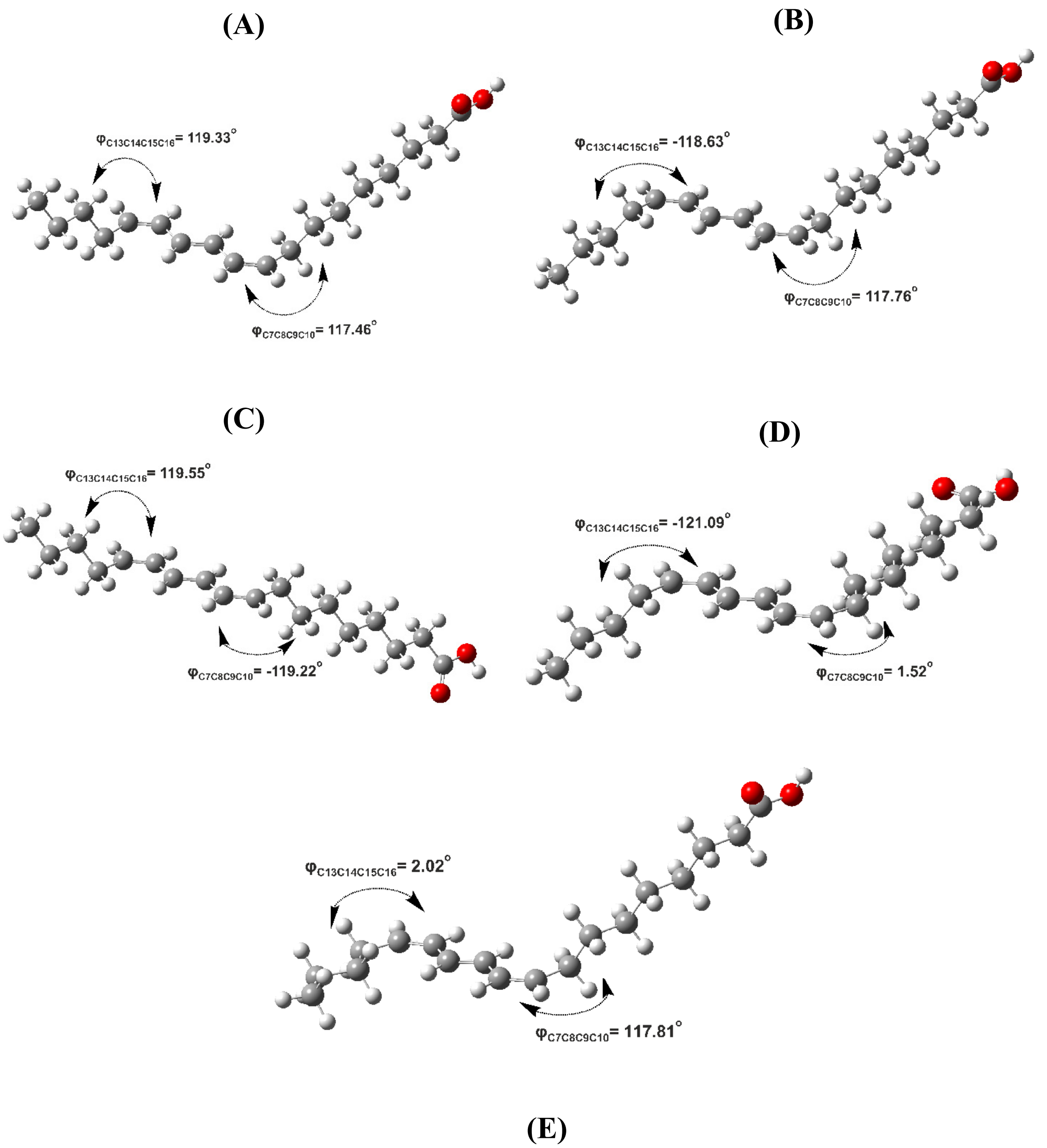

Figure S11. Structures of various conformers (**A**), (**B**), (**C**), (**D**) and (**E**) of the punicic acid (9*Z*,11*E*,13*Z*-isomer) with energy minimization in the gas-phase at the B3LYP/6-31+G(d) level of theory. ΔG values (kcal.mol^{-1}) and % populations of conformers (A), (B), (C), (D) and (E) are shown in Table 2.

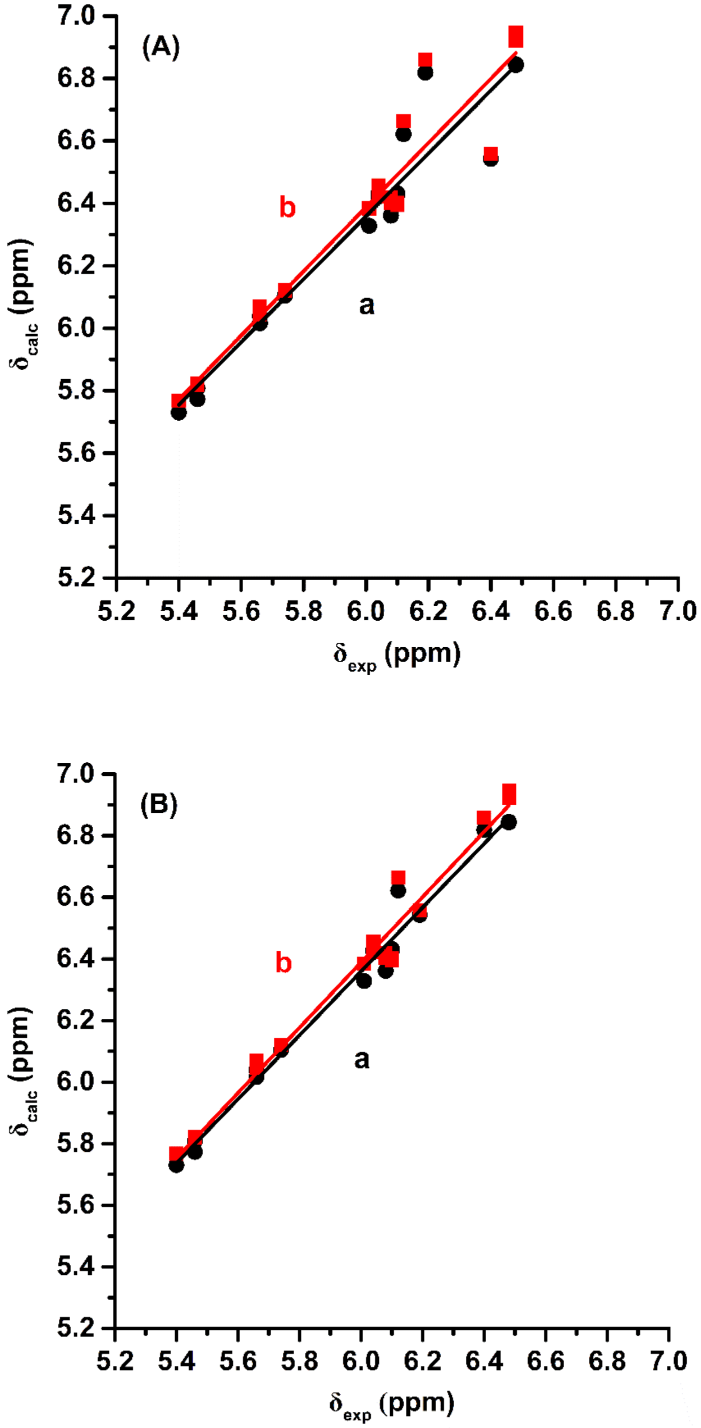

Figure S12. (A) Graphical presentation of calculated olefinic ^{1}H-NMR chemical shifts (at the GIAO/B3LYP/6-311+G(2d,p) (CPCM, $CHCl_3$) level), weighted by the respective Boltzmann factor of the various conformers of Table 2 (at the GIAO/B3LYP/6-311+G(2d,p) (CPCM, $CHCl_3$) level of theory *vs*. experimental values of the chemical shifts of the three 9,11,13-conjugated fatty acid geometrical isomers with optimization of the structures at the B3LYP/6-31+G(d) (**a**), and APFD/6-31+G(d) (**b**) level. **(B)** as in (A), but the literature experimental chemical shift assignment of H11 and H12 of β-eleostearic acid (9*Z*,11*E*,13*E* isomer) [18] has been revised.

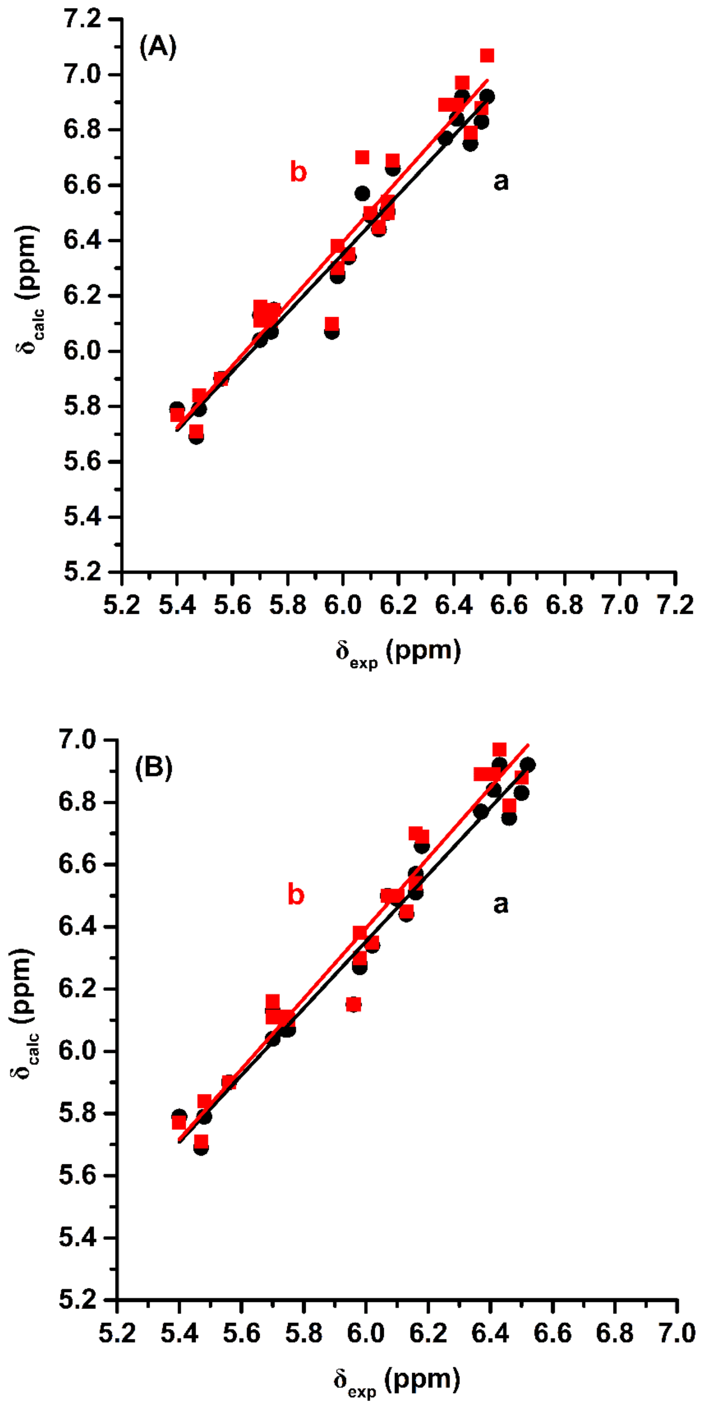

Figure S13 (A) Graphical presentation of calculated olefinic ^{1}H-NMR chemical shifts (at the GIAO/B3LYP/6-311+G(2d,p) (CPCM, $CHCl_3$) level), weighted by the respective Boltzmann factor of the various conformers of Table 2 *vs*. experimental values of the chemical shifts of the four 10,12,14-conjugated hexadecatrienyl acetate geometrical isomers with optimization of the structures at the B3LYP/6-31+G(d) (**a**), and APFD/6-31+G(d) (**b**) level. **(B)** as in (A), but the experimental chemical shifts of H13 and H15 of 9*Z*,11*Z*,13*E* [13] and of H13 and H14 of 9*Z*,11*E*,13*E*-conjugated hexadecatrienyl acetates [13] have been revised.

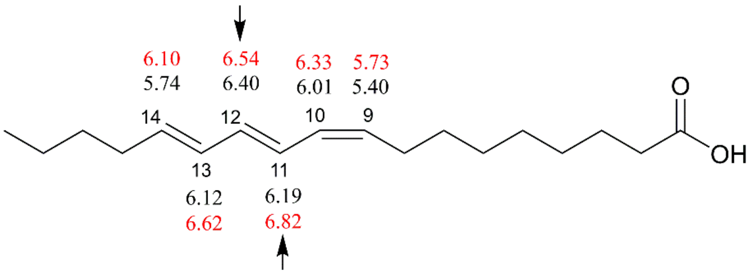

α-Eleostearic acid (9*Z*,11*E*,13*E* isomer)

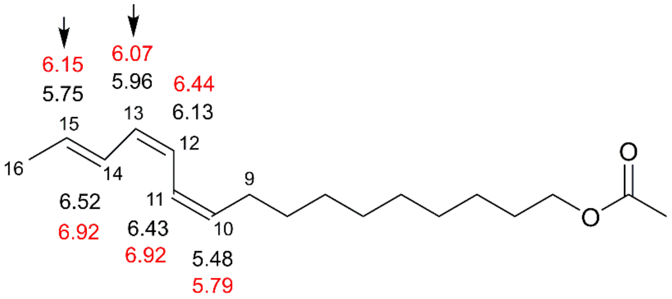

10*Z*,12*Z*,14*E*-Hexadecatrienyl Acetate

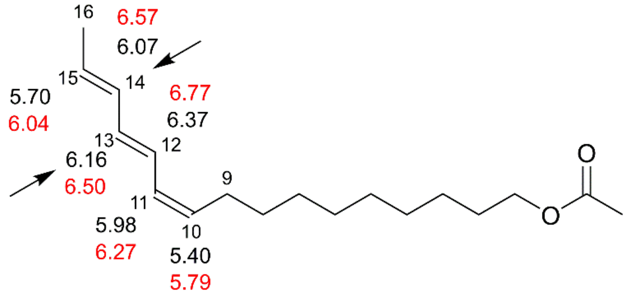

10*Z*,12*E*,14*E*-Hexadecatrienyl Acetate

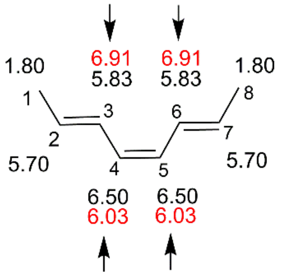

(*E*,*Z*,*E*)-2,4,6-Octatriene

Figure S14. Experimental ^{1}H NMR (δ_{exp}, black color) and calculated (δ_{calc}, red color) chemical shifts of the olefinic protons of (*E,Z,E*)-2,4,6-octatriene, α-eleostearic acid, (10*Z*, 12*Z*, 14*E*)-hexadecatrienyl acetate, and (10*Z*, 12*E*, 14*E*)-hexadecatrienyl acetate with optimization of the structures at the B3LYP/6-31+G(d) level of theory. The arrows denote the literature δ_{exp} values which should be revised.

Table S1. Experimental and computational (at the GIAO/B3LYP/6-311+G(2d,p) level with CPCM) ^{1}H-NMR chemical shifts of the model trienyl compounds (*Z*)-1,3,5-hexatriene, (*E*)-1,3,5-hexatriene, (*Z*)-1,3,5-hexatriene, (*E*)-,13,5-hexatriene, (*E,E,E*)-2,4,6-octatriene and (*E,Z,E*)-2,4,6-octatriene (Fig. 1) for various functionals and basis sets.

COMPOUND/ SOLVENT	GROUP	EXP	B3LYP/ 6-31 +G(d)	B3LYP/ 6-311 ++G(d,p)	B3LYP-D3/ 6-31 +G(d)	B3LYP-D3/ 6-311 ++G(d,p)	APFD/ 6-31 +G(d)	APFD/ 6-311 ++G(d,p)	PBE0/ 6-31 +G(d)	PBE0/ 6-311 ++G(d,p)	M062X/ 6-31 +G(d)	M062X/ 6-311 ++G(d,p)	ωB97XD/ 6-31 +G(d)	ωB97XD/ 6-311 ++G(d,p)
**(*Z*)-1,3,5-Hexatriene[a] ($CDCl_3$)**	C1-(H1a)	5.15	5.41	5.44	5.42	5.44	5.44	5.47	5.42	5.44	5.45	5.46	5.42	5.44
	C1-(H1b)	5.24	5.52	5.55	5.53	5.56	5.56	5.59	5.54	5.56	5.58	5.59	5.54	5.56
	C2	6.8	7.32	7.35	7.35	7.39	7.39	7.43	7.35	7.38	7.39	7.40	7.33	7.36
	C3	6.0	6.32	6.35	6.33	6.36	6.36	6.39	6.33	6.35	6.36	6.37	6.31	6.33
	C4	6.0	6.32	6.35	6.33	6.36	6.36	6.39	6.33	6.35	6.36	6.37	6.31	6.33
	C5	6.8	7.32	7.35	7.35	7.39	7.39	7.43	7.35	7.38	7.39	7.40	7.33	7.36
	C6-(H1a)	5.15	5.41	5.44	5.42	5.44	5.44	5.47	5.42	5.44	5.45	5.46	5.42	5.44
	C6(H1b)	5.24	5.52	5.55	5.53	5.56	5.56	5.59	5.54	5.56	5.58	5.59	5.54	5.56
**(*E*)-1,3,5-Hexatriene[a] ($CDCl_3$)**	C1-(H1a)	5.1	5.34	5.36	5.34	5.36	5.36	5.39	5.34	5.36	5.36	5.38	5.33	5.35
	C1-(H1b)	5.23	5.51	5.53	5.52	5.54	5.55	5.58	5.53	5.55	5.56	5.57	5.52	5.54
	C2	6.36	6.91	6.93	6.92	6.94	6.96	6.98	6.92	6.94	6.94	6.95	6.89	6.91
	C3	6.22	6.67	6.70	6.68	6.71	6.72	6.75	6.69	6.70	6.73	6.74	6.68	6.69
	C4	6.22	6.67	6.70	6.68	6.71	6.72	6.75	6.69	6.70	6.73	6.74	6.68	6.69
	C5	6.36	6.91	6.93	6.92	6.94	6.96	6.98	6.92	6.94	6.94	6.95	6.89	6.91
	C6-(H1a)	5.1	5.34	5.36	5.34	5.36	5.36	5.39	5.34	5.36	5.36	5.38	5.33	5.35

	C6(H1b)	5.23	5.51	5.53	5.52	5.54	5.55	5.58	5.53	5.55	5.56	5.57	5.52	5.54
**(*Z*)-1.3.5-Hexatriene[b,c] (CCl_4)**	C1-(H1a)	5.09	5.38	5.40	5.38	5.41	5.39	5.41	5.38	5.40	5.39	5.42	5.38	5.40
	C1-(H1b)	5.17	5.48	5.51	5.49	5.52	5.51	5.53	5.50	5.52	5.52	5.55	5.50	5.52
	C2	6.74	7.28	7.32	7.31	7.35	7.35	7.37	7.31	7.34	7.33	7.36	7.29	7.32
	C3	5.93	6.28	6.31	6.29	6.32	6.31	6.33	6.29	6.31	6.30	6.33	6.27	6.29
	C4	5.93	6.28	6.31	6.29	6.32	6.31	6.33	6.29	6.31	6.30	6.33	6.27	6.29
	C5	6.74	7.28	7.32	7.31	7.35	7.35	7.37	7.31	7.34	7.33	7.36	7.29	7.32
	C6-(H1a)	5.09	5.38	5.40	5.35	5.41	5.39	5.41	5.38	5.41	5.39	5.42	5.38	5.40
	C6(H1b)	5.17	5.48	5.51	5.49	5.52	5.51	5.53	5.50	5.52	5.52	5.55	5.50	5.52
**(*E*)-1.3.5-Hexatriene[b,c] (CCl_4)**	C1-(H1a)	5.05	5.30	5.33	5.30	5.33	5.32	5.34	5.31	5.32	5.31	5.34	5.29	5.32
	C1-(H1b)	5.18	5.47	5.50	5.48	5.51	5.50	5.52	5.49	5.51	5.50	5.53	5.48	5.51
	C2	6.3	6.87	6.90	6.88	6.91	6.91	6.92	6.88	6.90	6.88	6.91	6.85	6.87
	C3	6.16	6.63	6.65	6.64	6.67	6.67	6.69	6.64	6.66	6.67	6.70	6.63	6.65
	C4	6.16	6.63	6.65	6.64	6.67	6.67	6.69	6.64	6.66	6.67	6.70	6.63	6.65
	C5	6.3	6.87	6.90	6.88	6.91	6.91	6.92	6.88	6.90	6.88	6.91	6.85	6.87
	C6-(H1a)	5.05	5.30	5.33	5.30	5.33	5.32	5.34	5.31	5.32	5.31	5.34	5.29	5.32
	C6(H1b)	5.18	5.47	5.50	5.48	5.51	5.50	5.52	5.49	5.51	5.50	5.53	5.48	5.51
**(*E.Z.E*)-2.4.6-Octatriene[d] ($CDCl_3$)**	C1-(CH_3)	1.8	1.90	1.91	1.94	1.95	1.94	1.96	1.92	1.93	1.91	1.91	1.89	1.91
	C2-(CH)	5.7	6.07	6.10	6.08	6.11	6.12	6.14	6.08	6.10	6.13	6.15	6.08	6.10
	C3-(CH)	5.83	6.91	6.94	6.94	6.98	6.99	7.03	6.94	6.97	7.00	7.01	6.94	6.96

	C4-(CH)	6.5	6.03	6.06	6.03	6.07	6.07	6.09	6.03	6.05	6.07	6.08	6.02	6.04
	C5-(CH)	6.5	6.03	6.06	6.03	6.06	6.07	6.09	6.03	6.05	6.07	6.08	6.02	6.04
	C6-(CH)	5.83	6.91	6.94	6.94	6.98	6.99	7.03	6.94	6.97	7.00	7.01	6.94	6.96
	C7-(CH)	5.7	6.07	6.10	6.08	6.11	6.12	6.14	6.08	6.10	6.13	6.15	6.08	6.10
	C8-(CH)	1.8	1.90	1.91	1.94	1.95	1.94	1.96	1.92	1.93	1.91	1.91	1.89	1.91
**(*E.E.E*)-2.4.6-Octatriene[b] (CCl_4)**	C1-(CH_3)	1.84	1.86	1.87	1.86	1.89	1.88	1.89	1.87	1.88	1.84	1.86	1.84	1.85
	C2	5.69	5.98	6.00	5.98	6.05	6.00	6.02	5.98	6.00	6.01	6.04	5.98	6.00
	C3	6.12	6.45	6.48	6.46	6.38	6.49	6.50	6.46	6.47	6.47	6.50	6.44	6.45
	C4	6.10	6.55	6.58	6.56	6.19	6.59	6.61	6.57	6.58	6.59	6.62	6.55	6.58
	C5	6.10	6.55	6.58	6.56	6.19	6.59	6.61	6.57	6.58	6.59	6.62	6.55	6.58
	C6	612	6.45	6.48	6.46	6.38	6.49	6.50	6.46	6.47	6.47	6.50	6.44	6.45
	C7	5.69	5.98	6.00	5.98	6.05	6.00	6.02	5.98	6.00	6.01	6.04	5.98	6.00
	C8	1.84	1.86	1.87	1.86	1.89	1.88	1.89	1.87	1.88	1.84	1.86	1.84	1.85

[a] Ref. [15]; [b] Ref. [21]; [c] Ref. [22]; [d] Ref. [23]

Table S2. Experimental and computational (using the same level of theory as geometry optimization) ^{1}H-NMR chemical shifts of the model trienyl compounds (*Z*)-1,3,5-hexatriene, (*E*)-1,3,5-hexatriene, (*Z*)-1,3,5-hexatriene, (*E*)-,13,5-hexatriene, (*E,E,E*)-2,4,6-octatriene and (*E,Z,E*)-2,4,6-octatriene (Fig. 1) for various functionals and basis sets.

COMPOUND/ SOLVENT	GROUP	EXP	B3LYP/ 6-31 +G(d)	B3LYP/ 6-311 ++G(d.p)	B3LYP-D3/ 6-31 +G(d)	B3LYP-D3/ 6-311 ++G(d.p)	APFD/ 6-31 +G(d)	APFD/ 6-311 ++G(d.p)	PBE0/ 6-31 +G(d)	PBE0/ 6-311 ++G(d.p)	M062X/ 6-31 +G(d)	M062X/ 6-311 ++G(d.p)	*ω*B97XD /6-31 +G(d)	*ω*B97XD/ 6-311 ++G(d.p)
**(*Z*)-1.3.5-Hexatriene[a] ($CDCl_3$)**	C1-(H1a)	5.15	5.27	5.46	5.27	5.47	5.39	5.57	5.40	5.57	5.89	6.07	5.43	5.43
	C1-(H1b)	5.24	5.34	5.57	5.34	5.58	5.46	5.68	5.47	5.68	5.94	6.13	5.51	5.51
	C2	6.8	7.12	7.37	7.14	7.39	7.25	7.47	7.24	7.45	7.82	7.99	7.20	7.20
	C3	6.0	6.13	6.34	6.14	6.35	6.26	6.44	6.26	6.42	6.64	6.81	6.24	6.24
	C4	6.0	6.13	6.34	6.14	6.35	6.26	6.44	6.26	6.42	6.64	6.82	6.24	6.24
	C5	6.8	7.12	7.37	7.14	7.39	7.25	7.47	7.24	7.45	7.82	7.99	7.20	7.20
	C6-(H1a)	5.15	5.27	5.46	5.27	5.47	5.39	5.57	5.39	5.57	5.89	6.07	5.42	5.42
	C6(H1b)	5.24	5.34	5.57	5.34	5.58	5.46	5.68	5.47	5.68	5.94	6.13	5.51	5.51
**(*E*)-1.3.5-Hexatriene[a] ($CDCl_3$)**	C1-(H1a)	5.1	5.21	5.39	5.21	5.39	5.33	5.50	5.34	5.50	5.73	5.87	5.32	5.32
	C1-(H1b)	5.23	5.35	5.57	5.35	5.58	5.47	5.68	5.48	5.68	5.90	6.13	5.47	5.47
	C2	6.36	6.66	6.94	6.67	6.95	6.78	7.01	6.78	7.00	7.29	7.49	6.75	6.75
	C3	6.22	6.23	6.65	6.24	6.65	6.38	6.73	6.38	6.72	6.91	7.25	6.48	6.48
	C4	6.22	6.23	6.65	6.24	6.65	6.38	6.73	6.38	6.72	6.91	7.25	6.48	6.48
	C5	6.36	6.66	6.94	6.67	6.95	6.78	7.01	6.78	7.00	7.29	7.49	6.75	6.75
	C6-(H1a)	5.1	5.21	5.39	5.35	5.58	5.33	5.50	5.34	5.50	5.73	5.87	5.32	5.32

	C6(H1b)	5.23	5.35	5.57	5.21	5.39	5.47	5.68	5.48	5.68	5.90	6.13	5.47	5.47
**(*Z*)-1.3.5-Hexatriene[b.c] (CCl_4)**	C1-(H1a)	5.09	5.23	5.42	5.23	5.42	5.36	5.52	5.35	5.53	5.84	6.02	5.38	5.38
	C1-(H1b)	5.17	5.29	5.52	5.30	5.54	5.43	5.63	5.42	5.63	5.89	6.08	5.46	5.46
	C2	6.74	7.08	7.32	7.09	7.35	7.21	7.42	7.19	7.41	7.77	7.95	7.16	7.16
	C3	5.93	6.09	6.30	6.10	6.31	6.23	6.39	6.21	6.38	6.60	6.77	6.19	6.19
	C4	5.93	6.09	6.30	6.10	6.31	6.23	6.39	6.21	6.38	6.60	6.77	6.19	6.19
	C5	6.74	7.08	7.32	7.09	7.35	7.21	7.42	7.19	7.41	7.77	7.95	7.16	7.16
	C6-(H1a)	5.09	5.23	5.42	5.23	5.42	5.35	5.52	5.35	5.53	5.84	6.02	5.38	5.38
	C6(H1b)	5.17	5.29	5.53	5.30	5.54	5.42	5.63	5.42	5.63	5.89	6.08	5.46	5.46
**(*E*)-1.3.5-Hexatriene[b.c] (CCl_4)**	C1-(H1a)	5.05	5.17	5.35	5.17	5.35	5.30	5.46	5.29	5.46	5.68	5.83	5.28	5.28
	C1-(H1b)	5.18	5.31	5.53	5.31	5.54	5.44	5.63	5.43	5.63	5.85	6.08	5.43	5.43
	C2	6.3	6.62	6.90	6.63	6.91	6.75	6.97	6.73	6.96	7.24	7.45	6.71	6.71
	C3	6.16	6.19	6.60	6.19	6.61	6.34	6.68	6.33	6.67	6.86	7.20	6.43	6.43
	C4	6.16	6.19	6.60	6.19	6.61	6.34	6.68	6.33	6.67	6.86	7.20	6.43	6.43
	C5	6.3	6.62	6.90	6.63	6.91	6.75	6.97	6.73	6.96	7.24	7.45	6.71	6.71
	C6-(H1a)	5.05	5.17	5.35	5.31	5.54	5.30	5.46	5.29	5.46	5.68	5.83	5.28	5.28
	C6(H1b)	5.18	5.31	5.53	5.17	5.35	5.44	5.63	5.43	5.63	5.85	6.08	5.43	5.43
**(*E.Z.E*)-2.4.6-Octatriene[d] ($CDCl_3$)**	C1-(CH_3)	1.8	1.87	1.92	1.89	1.96	1.88	1.93	1.78	1.92	2.10	2.04	1.60	1.60
	C2-(CH)	5.7	5.84	6.07	5.84	6.08	5.92	6.14	5.83	6.13	6.30	6.47	6.03	6.03
	C3-(CH)	5.83	6.79	6.99	6.81	7.02	6.90	7.08	6.81	7.06	7.42	7.74	6.74	6.74

	C4-(CH)	6.5	5.87	6.09	5.88	6.09	5.99	6.18	5.89	6.17	6.33	6.45	6.05	6.05
	C5-(CH)	6.5	5.87	6.09	5.88	6.09	5.99	6.18	5.89	6.17	6.33	6.45	6.05	6.05
	C6-(CH)	5.83	6.79	6.99	6.81	7.02	6.90	7.08	6.81	7.06	7.42	7.74	6.74	6.74
	C7-(CH)	5.7	584	6.07	5.84	6.08	5.92	6.14	5.83	6.13	6.30	6.47	6.03	6.03
	C8-(CH)	1.8	1.87	1.92	1.89	1.96	1.88	1.93	1.78	1.92	2.10	2.04	1.60	1.60
**(*E.E.E*)-2.4.6-Octatriene[b] (CCl_4)**	C1-(CH_3)	1.84	1.85	1.90	1.86	1.90	1.85	1.90	1.84	1.89	1.79	1.84	1.75	1.75
	C2	5.69	5.93	6.07	5.93	5.99	6.01	6.13	6.00	6.12	6.43	6.50	5.97	5.97
	C3	6.12	6.07	6.49	6.07	6.39	6.20	6.54	6.19	6.53	6.77	7.17	6.23	6.23
	C4	6.10	5.85	6.42	5.85	6.38	6.01	6.49	6.01	6.48	6.51	7.07	6.18	6.18
	C5	6.10	5.85	6.42	5.85	6.38	6.01	6.49	6.01	6.48	6.51	7.07	6.18	6.18
	C6	612	6.07	6.49	6.07	6.39	6.20	6.54	6.19	6.53	6.77	7.17	6.23	6.23
	C7	5.69	5.93	6.07	5.93	5.99	6.01	6.13	6.00	6.12	6.43	6.50	5.97	5.97
	C8	1.84	1.85	1.90	1.86	1.90	1.85	1.90	1.84	1.89	1.79	1.84	1.75	1.75

[a] Ref. [15]; [b] Ref. [21]; [c] Ref. [22]; [d] Ref. [23]

Table S3. Linear regression correlation coefficient, mean square error, intercept and slope of calculated (at the GIAO/B3LYP/6-311+G(2d,p) level with CPCM) *vs*. experimental ^{1}H chemical shifts of the model trienyl compounds (*Z*)-1,3,5-hexatriene, (*E*)-1,3,5-hexatriene, (*E,Z,E*)-2,4,6-octatriene and (*E,E,E*)-2,4,6-octatriene determined from various optimized geometries. (A) Data of Fig. S2(A); (B) data of Fig. S2(B).

	**(A)**				**(B)**			
**Method**	**Correlation coefficient R^2**	**Mean square error**	**Intercept**	**Slope**	**Correlation coefficient R^2**	**Mean square error**	**Intercept**	**Slope**
B3LYP/6-31+G(d)	0.847	0.067	-0.067	1.074	0.991	0.004	-0.561	1.160
B3LYP/6-311++G(d,p)	0.848	0.067	-0.056	1.077	0.991	0.004	-0.550	1.162
B3LYP-D3/6-31+G(d)	0.844	0.067	-0.108	1.083	0.991	0.004	-0.619	1.171
B3LYP-D3/6-311++G(d,p)	0.827	0.076	0.012	1.064	0.975	0.011	-0.502	1.153
APFD/6-31+G(d)	0.844	0.071	-0.142	1.094	0.991	0.004	-0.658	1.183
APFD/6-311++G(d,p)	0.841	0.072	-0.113	1.093	0.991	0.004	-0.641	1.184
PBE0/6-31+G(d)	0.843	0.070	-0.078	1.079	0.990	0.004	-0.588	1.167
PBE0/6-311++G(d,p)	0.842	0.071	-0.072	1.081	0.990	0.004	-0.589	1.170
M06-2X/6-31+G(d)	0.841	0.071	-0.082	1.084	0.991	0.004	-0.604	1.174
M06-2X/6-311++G(d,p)	0.841	0.071	-0.049	1.082	0.991	0.004	-0.571	1.172
*ω*B97XD/6-31+G(d)	0.840	0.070	-0.031	1.069	0.990	0.004	-0.547	1.158
*ω*B97XD/6-311++G(d,p)	0.840	0.070	-0.011	1.069	0.990	0.004	-0.527	1.158

Table S4. (**A**) Linear regression correlation coefficient, mean square error, intercept and slope of calculated *vs*. experimental ^{1}H chemical shifts of the olefinic protons of the model trienyl compounds (*Z*)-1,3,5-hexatriene, (*E*)-1,3,5-hexatriene, (*E,Z,E*)-2,4,6-octatriene and (*E,E,E*)-2,4,6-octatriene using the same level of theory as geometry optimization (Fig. 2(A)). (**B**) As in (A), but the literature experimental values of δ(H(3,6)) = 5.83 ppm and δ(H (4,5)) = 6.50 ppm, of (*E,Z,E*)-2,4,6-octatriene [23] have been reversed.

	**(A)**				**(B)**			
**Method**	Correlation coefficient R^2	Mean square error	Intercept	Slope	Correlation coefficient R^2	Mean square error	Intercept	Slope
B3LYP/6-31+G(d)	0.799	0.077	0.257	0.979	0.955	0.017	-0.258	1.068
B3LYP/6-311++G(d,p)	0.840	0.067	0.105	1.050	0.990	0.004	-0.401	1.137
B3LYP-D3/6-31+G(d)	0.794	0.080	0.241	0.983	0.950	0.020	-0.279	1.072
B3LYP-D3/6-311++G(d,p)	0.827	0.074	0.110	1.049	0.978	0.009	-0.409	1.139
APFD/6-31+G(d)	0.806	0.075	0.353	0.984	0.961	0.015	-0.159	1.073
APFD/6-311++G(d,p)	0.837	0.067	0.267	1.038	0.988	0.005	-0.239	1.125
PBE0/6-31+G(d)	0.800	0.075	0.419	0.970	0.957	0.016	-0.094	1.058
PBE0/6-311++G(d,p)	0.837	0.066	0.326	1.026	0.987	0.005	-0.172	1.112
M06-2X/6-31+G(d)	0.786	0.093	0.532	1.033	0.958	0.018	-0.077	1.138
M06-2X/6-311++G(d,p)	0.773	0.108	0.527	1.073	0.970	0.014	-0.200	1.198
*ω*B97XD/6-31+G(d)	0.858	0.051	0.389	0.980	0.982	0.006	0.003	1.046
*ω*B97XD/6-311++G(d,p)	0.828	0.062	0.674	0.963	0.984	0.006	0.185	1.048

Table S5. Effect of variation of the torsion angle φ($_1C_2C_3C_4$) of (*Z*)-1.3.5-hexatriene, with energy minimization at the B3LYP/6-31+G(d) level on calculated ^{1}H-NMR chemical shifts (at the GIAO/B3LYP/6-311+G(2d.p) level with CPCM in $CHCl_3$).

Degrees	H1a	H1b	H2	H3	H4	H5	H6a	H6b
**0**	5.53	5.94	6.55	5.85	6.40	7.55	5.59	5.66
**10**	5.56	5.88	6.57	5.96	6.48	7.50	5.55	5.68
**20**	5.60	5.78	6.57	6.04	6.51	7.46	5.50	5.68
**30**	5.62	5.69	6.58	6.11	6.52	7.43	5.45	5.65
**40**	5.62	5.60	6.59	6.18	6.51	7.41	5.40	5.62
**50**	5.60	5.52	6.59	6.24	6.49	7.36	5.36	5.59
**60**	5.58	5.45	6.63	6.29	6.47	7.30	5.34	5.56
**70**	5.55	5.40	6.68	6.32	6.45	7.22	5.32	5.53
**80**	5.54	5.37	6.72	6.35	6.43	7.12	5.32	5.50
**90**	5.54	5.34	6.81	6.41	6.43	7.04	5.33	5.49
**100**	5.55	5.33	6.91	6.47	6.42	6.97	5.34	5.48
**110**	5.55	5.35	7.01	6.52	6.42	6.95	5.36	5.47
**120**	5.53	5.37	7.13	6.52	6.40	7.01	5.37	5.47
**130**	5.49	5.41	7.20	6.47	6.38	7.10	5.37	5.47
**140**	5.46	5.44	7.28	6.43	6.36	7.19	5.38	5.48
**150**	5.43	5.47	7.33	6.39	6.35	7.25	5.39	5.50
**160**	5.41	5.49	7.36	6.36	6.34	7.29	5.40	5.51
**170**	5.41	5.52	7.35	6.33	6.33	7.31	5.41	5.52
**180**	5.42	5.52	7.32	6.32	6.32	7.32	5.41	5.52

Table S6. Effect of variation of the torsion angle φ($C_1C_2C_3C_4$) of (*Z*)-1.3.5-hexatriene, on calculated ^{1}H-NMR chemical shifts at the same level of theory as geometry optimization (APFD/6-311++G(d,p) with CPCM in $CHCl_3$).

Degrees	H1a	H1b	H2	H3	H4	H5	H6a	H6b
**0**	5.74	6.20	6.66	5.96	6.57	7.79	5.76	5.81
**10**	5.79	6.11	6.72	6.12	6.70	7.68	5.72	5.86
**20**	5.83	5.99	6.72	6.21	6.72	7.61	5.65	5.86
**30**	5.84	5.88	6.71	6.30	6.71	7.55	5.59	5.84
**40**	5.84	5.78	6.71	6.37	6.68	7.51	5.54	5.80
**50**	5.82	5.70	6.72	6.43	6.65	7.46	5.51	5.76
**60**	5.79	5.63	6.75	6.47	6.62	7.41	5.48	5.72
**70**	5.76	5.58	6.80	6.50	6.59	7.33	5.47	5.69
**80**	5.75	5.55	6.85	6.52	6.55	7.22	5.46	5.65
**90**	5.74	5.51	6.92	6.56	6.54	7.16	5.48	5.64
**100**	5.75	5.51	7.01	6.60	6.54	7.10	5.50	5.64
**110**	5.76	5.52	7.14	6.64	6.52	7.07	5.51	5.63
**120**	5.72	5.55	7.26	6.64	6.51	7.14	5.53	5.64
**130**	5.71	5.58	7.33	6.59	6.49	7.22	5.53	5.65
**140**	5.66	5.63	7.41	6.54	6.48	7.32	5.55	5.67
**150**	5.62	5.66	7.46	6.50	6.47	7.39	5.55	5.68
**160**	5.60	5.69	7.48	6.53	6.46	7.42	5.56	5.68
**170**	5.58	5.70	7.49	5.44	6.44	7.46	5.56	5.67
**180**	5.57	5.68	7.47	6.44	6.44	7.47	5.57	5.68

Table S7. Effect of variation of the $C_2C_3C_4C_5$ torsion angle of (*E.Z.E*)-2.4.6-octatriene on calculated ^{1}H-NMR chemical shifts (at the GIAO/B3LYP/6-311+G(2d,p) level of theory with CPCM in $CHCl_3$).

Degrees	H1	H2	H3	H4	H5	H6	H7	H8
**0**	1.94	6.67	6.17	5.52	6.14	7.13	6.22	1.97
**10**	1.94	6.61	6.18	5.60	6.27	7.07	6.23	1.97
**20**	1.94	6.48	6.16	5.70	6.29	7.03	6.23	1.95
**30**	1.94	6.35	6.17	5.77	6.30	7.00	6.21	1.93
**40**	1.93	6.21	6.16	5.83	6.31	6.96	6.18	1.91
**50**	1.91	6.09	6.15	5.90	6.31	6.92	6.15	1.89
**60**	1.88	6.01	6.21	5.95	6.31	6.89	6.13	1.88
**70**	1.87	5.93	6.23	6.00	6.31	6.82	6.10	1.87
**80**	1.87	5.85	6.25	6.04	6.32	6.72	6.08	1.86
**90**	1.85	5.84	6.36	6.12	6.32	6.67	6.07	1.86
**100**	1.85	5.85	6.46	6.18	6.31	6.61	6.07	1.87
**110**	1.86	5.85	6.56	6.22	6.29	6.59	6.06	1.88
**120**	1.86	5.92	6.70	6.22	6.23	6.66	6.06	1.89
**130**	1.89	5.93	6.76	6.17	6.19	6.74	6.06	1.90
**140**	1.90	5.98	6.86	6.12	6.17	6.83	6.06	1.91
**150**	1.90	6.03	6.94	6.09	6.14	6.89	6.05	1.92
**160**	1.91	6.06	6.97	6.08	6.11	6.90	6.04	1.92
**170**	1.92	6.09	6.97	6.04	6.05	6.90	6.04	1.93
**180**	1.93	6.07	6.91	6.03	6.03	6.91	6.07	1.93

Table S8. Effect of variation of the $C_2C_3C_4C_5$ torsion angle of (*E.Z.E*)-2.4.6-octatriene, on calculated ^{1}H-NMR chemical shifts at the same level of theory as geometry optimization (APFD/6-311++G(d,p) with CPCM in $CHCl_3$).

Degrees	H1	H2	H3	H4	H5	H6	H7	H8
**0**	1.94	6.87	6.31	5.63	6.31	7.37	6.30	2.01
**10**	1.99	6.80	6.34	5.79	6.46	7.27	6.35	2.01
**20**	2.00	6.65	6.31	5.89	6.49	7.20	6.36	1.98
**30**	2.00	6.49	6.31	5.97	6.49	7.13	6.34	1.96
**40**	1.99	6.36	6.29	6.04	6.49	7.09	6.30	1.93
**50**	1.98	6.23	6.30	6.10	6.47	7.05	6.27	1.92
**60**	1.94	6.12	6.33	6.15	6.47	7.04	6.24	1.91
**70**	1.92	6.03	6.36	6.17	6.46	6.96	6.21	1.90
**80**	1.93	5.96	6.41	6.19	6.44	6.87	6.19	1.89
**90**	1.91	5.94	6.48	6.24	6.44	6.83	6.18	1.90
**100**	1.90	5.95	6.58	6.29	6.43	6.76	6.18	1.91
**110**	1.92	5.96	6.70	6.33	6.40	6.73	6.18	1.92
**120**	1.90	6.02	6.83	6.34	6.36	6.78	6.19	1.94
**130**	1.94	6.06	6.91	6.29	6.32	6.86	6.19	1.94
**140**	1.95	6.11	7.01	6.24	6.29	6.94	6.20	1.96
**150**	1.95	6.15	7.08	6.21	6.26	7.01	6.19	1.96
**160**	1.97	6.19	7.10	6.18	6.23	7.03	6.16	1.96
**170**	1.97	6.23	7.12	6.14	6.18	7.06	6.14	1.95
**180**	1.95	6.14	7.08	6.18	6.18	7.08	6.14	1.95

Table S9. Effect of variation of the $C_1C_2C_3C_4$ torsion angle of (*E*)-1.3.5-hexatriene on calculated ^{1}H-NMR chemical shifts (at the GIAO/B3LYP/6-311+G(2d.p) level with CPCM in $CHCl_3$).

Degrees	H1a	H1b	H2	H3	H4	H5	H6a	H6b
**0**	5.33	6.08	6.55	6.30	7.30	6.87	5.31	5.54
**10**	5.31	6.04	6.57	6.32	7.24	6.85	5.33	5.54
**20**	5.28	5.94	6.62	6.37	7.12	6.81	5.35	5.54
**30**	5.25	5.80	6.68	6.44	6.97	6.79	5.35	5.54
**40**	5.24	5.66	6.74	6.50	6.81	6.78	5.34	5.52
**50**	5.25	5.54	6.79	6.55	6.68	6.77	5.33	5.50
**60**	5.28	5.43	6.82	6.58	6.57	6.76	5.32	5.48
**70**	5.31	5.36	6.84	6.59	6.48	6.75	5.32	5.47
**80**	5.36	5.32	6.88	6.62	6.43	6.75	5.31	5.45
**90**	5.42	5.29	6.93	6.68	6.41	6.75	5.30	5.44
**100**	5.47	5.31	6.95	6.74	6.38	6.75	5.29	5.43
**110**	5.51	5.31	6.98	6.79	6.41	6.79	5.30	5.42
**120**	5.48	5.34	6.95	6.75	6.41	6.77	5.31	5.42
**130**	5.44	5.37	6.95	6.72	6.44	6.78	5.33	5.44
**140**	5.40	5.42	6.92	6.69	6.50	6.79	5.34	5.46
**150**	5.38	5.45	6.86	6.67	6.56	6.81	5.35	5.48
**160**	5.37	5.48	6.83	6.66	6.62	6.85	5.34	5.50
**170**	5.35	5.50	6.87	6.67	6.66	6.89	5.34	5.51
**180**	5.34	5.51	6.91	6.67	6.67	6.91	5.34	5.51

Table S10. Effect of variation of the $C_1C_2C_3C_4$ torsion angle of (*E*)-1.3.5-hexatriene, on calculated ^{1}H-NMR chemical shifts at the same level of theory as geometry optimization (APFD/6-311++G(d,p) with CPCM in $CHCl_3$)

Degrees	H1a	H1b	H2	H3	H4	H5	H6a	H6b
**0**	5.53	6.26	6.63	6.40	7.42	6.97	5.48	5.73
**10**	5.51	6.23	6.66	6.42	7.37	6.96	5.50	5.74
**20**	5.48	6.13	6.74	6.49	7.26	6.95	5.51	5.73
**30**	5.44	5.99	6.81	6.57	7.10	6.95	5.50	5.71
**40**	5.44	5.84	6.87	6.64	6.94	6.94	5.49	5.69
**50**	5.45	5.70	6.93	6.68	6.79	6.93	5.48	5.67
**60**	5.48	5.59	6.96	6.71	6.67	6.92	5.47	5.65
**70**	5.52	5.52	6.97	6.74	6.58	6.90	5.46	5.63
**80**	5.54	5.49	7.00	6.76	6.53	6.90	5.46	5.61
**90**	5.59	5.47	7.04	6.81	6.51	6.90	5.45	5.60
**100**	5.68	5.47	7.06	6.85	6.49	6.90	5.44	5.59
**110**	5.72	5.49	7.08	6.88	6.48	6.91	5.46	5.59
**120**	5.70	5.50	7.06	6.88	6.53	6.91	5.47	5.59
**130**	5.62	5.56	7.06	6.85	6.58	6.92	5.48	5.62
**140**	5.57	5.61	7.03	6.82	6.62	6.93	5.49	5.64
**150**	5.56	5.64	6.99	6.79	6.67	6.95	5.50	5.66
**160**	5.56	5.65	6.97	6.77	6.71	6.97	5.51	5.68
**170**	5.52	5.67	6.99	6.74	6.73	7.00	5.51	5.69
**180**	5.50	5.68	7.01	6.73	6.73	7.01	5.50	5.68

Table S11. Effect of variation of the $C_2C_3C_4C_5$ torsion angle of (*E.E.E*)-2.4.6-octatriene on calculated ^{1}H-NMR chemical shifts (at the GIAO/B3LYP/6-311+G(2d,p) level with CPCM in CCl_4).

Degrees	H1	H2	H3	H4	H5	H6	H7	H8
**0**	1.86	6.56	6.13	5.96	6.93	6.44	6.04	1.87
**10**	1.85	6.45	6.16	6.07	6.84	6.40	6.04	1.87
**20**	1.84	6.31	6.20	6.16	6.73	6.37	6.03	1.87
**30**	1.83	6.16	6.26	6.23	6.60	6.34	6.02	1.86
**40**	1.82	6.03	6.31	6.25	6.49	6.33	6.00	1.85
**50**	1.81	5.93	6.33	6.26	6.40	6.33	5.98	1.85
**60**	1.80	5.86	6.33	6.26	6.32	6.32	5.97	1.85
**70**	1.78	5.82	6.39	6.30	6.29	6.32	5.97	1.85
**80**	1.74	5.83	6.50	6.38	6.29	6.33	5.97	1.85
**90**	1.79	5.79	6.45	6.42	6.25	6.36	5.97	1.84
**100**	1.77	5.84	6.57	6.50	6.22	6.33	5.97	1.84
**110**	1.83	5.80	6.47	6.52	6.26	6.40	5.98	1.84
**120**	1.81	5.89	6.52	6.44	6.27	6.41	5.96	1.85
**130**	1.82	5.96	6.51	6.42	6.31	6.40	5.97	1.85
**140**	1.85	5.93	6.40	6.44	6.39	6.41	5.98	1.86
**150**	1.86	5.93	6.35	6.47	6.47	6.42	6.00	1.86
**160**	1.85	5.97	6.39	6.51	6.53	6.44	5.99	1.85
**170**	1.86	5.98	6.45	6.55	6.55	6.45	5.98	1.85
**180**	1.86	6.56	6.13	5.96	6.93	6.44	6.04	1.87

Table S12. Effect of variation of the $C_2C_3C_4C_5$ torsion angle of (*E.E.E*)-2.4.6-octatriene, on calculated ^{1}H-NMR chemical shifts at the same level of theory as geometry optimization (APFD/6-311++G(d.p) with CPCM in CCl_4).

Degrees	H1	H2	H3	H4	H5	H6	H7	H8
**0**	1.87	6.70	6.22	6.03	7.12	6.52	6.13	1.89
**10**	1.91	6.67	6.26	6.09	7.06	6.56	6.15	1.91
**20**	1.90	6.57	6.29	6.18	6.97	6.55	6.16	1.91
**30**	1.89	6.44	6.34	6.27	6.85	6.53	6.16	1.90
**40**	1.88	6.28	6.40	6.33	6.72	6.51	6.14	1.89
**50**	1.87	6.14	6.46	6.36	6.60	6.49	6.11	1.88
**60**	1.87	6.03	6.47	6.37	6.50	6.48	6.10	1.88
**70**	1.86	5.97	6.45	6.39	6.42	6.47	6.09	1.87
**80**	1.83	5.94	6.48	6.42	6.39	6.47	6.08	1.87
**90**	1.78	5.93	6.57	6.47	6.40	6.49	6.08	1.88
**100**	1.84	5.90	6.57	6.51	6.37	6.51	6.06	1.87
**110**	1.81	5.94	6.65	6.60	6.35	6.49	6.07	1.87
**120**	1.89	5.94	6.61	6.60	6.33	6.48	6.07	1.89
**130**	1.86	5.98	6.62	6.58	6.38	6.51	6.07	1.89
**140**	1.86	6.04	6.61	6.56	6.40	6.50	6.08	1.89
**150**	1.90	6.04	6.55	6.55	6.44	6.50	6.10	1.89
**160**	1.91	6.05	6.51	6.54	6.49	6.51	6.13	1.90
**170**	1.89	6.11	6.52	6.51	6.50	6.52	6.14	1.90
**180**	1.90	6.13	6.54	6.48	6.48	6.54	6.13	1.90

Table S13. Calculated (δ_{calc}, ppm) and experimental (δ_{exp}, ppm) ^{1}H-NMR chemical shifts of the 9,11,13-conjugated fatty acid geometric isomers with geometry optimization at the B3LYP/6-31+G(d) and APFD/6-31+G(d) level.

Compound	Atom	$\delta_{exp.}$ (ppm)[a]	B3LYP/6-31+G(d) δ_{calc} (ppm)[b]	APFD/6-31+G(d) δ_{calc} (ppm)[b]
9*E*,11*E*,13*E* Isomer (β-Eleostearic acid)	H11	6.10	6.43	6.40
	H12	6.10	6.43	6.39
	H10	6.04	6.42	6.42
	H13	6.04	6.43	6.46
	H9	5.66	6.02	6.05
	H14	5.66	6.04	6.07
	H2	2.37	2.47	2.48
	H8	2.10	2.13	2.15
	H15	2.10	2.13	2.14
	H3	1.65	1.48	1.51
	H4	1.39	1.24	1.25
	H5	1.39	1.31	1.33
	H6	1.39	1.30	1.32
	H7	1.39	1.28	1.29
	H16	1.33	1.23	1.24
	H17	1.33	1.36	1.37
	H18	0.91	0.93	0.96
9*Z*,11*E*,13*E* Isomer (β-Eleostearic acid)	H11	6.19	6.82	6.86
	H12	6.40	6.54	6.56
	H10	6.01	6.33	6.38
	H13	6.12	6.62	6.66
	H9	5.4	5.73	5.77
	H14	5.74	6.10	6.12
	H2	2.40	2.48	2.51
	H8	2.20	2.26	2.27
	H15	2.20	2.10	2.11
	H3	1.64	1.48	1.50
	H4	1.41	1.25	1.26
	H5	1.41	1.32	1.34
	H6	1.41	1.36	1.37
	H7	1.41	1.26	1.25
	H16	1.33	1.27	1.28
	H17	1.33	1.36	1.37
	H18	0.97	0.97	0.98
9*Z*,11*E*,13*Z* Isomer (Punicic acid)	H11	6.48	6.85	6.95
	H12	6.48	6.84	6.92
	H10	6.08	6.36	6.40
	H13	6.08	6.41	6.42
	H9	5.46	5.81	5.82
	H14	5.46	5.77	5.82
	H2	2.37	2.49	2.50
	H8	2.22	2.30	2.29
	H15	2.22	2.29	2.29
	H3	1.65	1.50	1.51
	H4	1.39	1.25	1.26
	H5	1.39	1.32	1.32
	H6	1.39	1.35	1.37
	H7	1.39	1.29	1.27

H16	1.32	1.24	1.22
H17	1.32	1.41	1.42
H18	0.94	0.97	0.98

[a] Ref. [18]; [b] at the GIAO/B3LYP/6-311+G(2d,p) level with CPCM.

Table S14. Statistical analysis of the data of Figure S12 and Table S13.

	R^2	Slope	Intercept
(A) (a)	0.922	1.008	0.312
(A) (b)	0.910	1.028	0.219
(B) (a)	0.982	1.038	0.133
(B) (b)	0.972	1.061	0.024

Table S15. Calculated (δ_{calc}, ppm) and experimental (δ_{exp}, ppm) ^{1}H-NMR chemical shifts of the 10,12,14-conjugated hexadecatrienyl acetate geometric isomers with geometry optimization at the B3LYP/6-31+G(d) and APFD/6-31+G(d) level.

Compound	Atom	$\delta_{exp.}$ (ppm)[a]	B3LYP/6-31+G(d) δ_{calc} (ppm)[b]	APFD/6-31+G(d) δ_{calc} (ppm)[b]
10*E*,12*E*,14*Z*-Hexadecatrienyl acetate	H9	2.09	2.16	2.16
	H10	5.70	6.13	6.16
	H11	6.10	6.49	6.50
	H12	6.18	6.66	6.69
	H13	6.41	6.84	6.89
	H14	6.02	6.34	6.35
	H15	5.47	5.69	5.71
	H16	1.76	1.92	1.93
10*E*,12*Z*,14*Z*-Hexadecatrienyl acetate	H9	2.12	2.21	2.22
	H10	5.74	6.07	6.11
	H11	6.50	6.83	6.88
	H12	5.98	6.28	6.30
	H13	6.16	6.51	6.54
	H14	6.46	6.75	6.79
	H15	5.56	5.90	5.90
	H16	1.77	1.90	1.90
10*Z*,12*Z*,14*E*-Hexadecatrienyl acetate	H9	2.18	2.25	2.27
	H10	5.48	5.79	5.84
	H11	6.43	6.92	6.97
	H12	6.13	6.44	6.45
	H13	5.96	6.07	6.10
	H14	6.52	6.92	7.07
	H15	5.75	6.15	6.15
	H16	1.80	1.93	1.94
10*Z*,12*E*,14*E*-Hexadecatrienyl acetate	H9	2.17	2.31	2.30
	H10	5.40	5.79	5.77
	H11	5.98	6.27	6.38
	H12	6.37	6.77	6.89
	H13	6.16	6.50	6.50
	H14	6.07	6.57	6.70

	H15	5.70	6.04	6.11
	H16	1.77	1.90	1.90

[a] Ref. [13]; [b] at the GIAO/B3LYP/6-311+G(2d,p) level with CPCM.

Table S16. Statistical analysis of the data of Figure S13 and Table S15.

	R^2	Slope	Intercept
(A) (a)	0.949	1.067	-0.046
(A) (b)	0.938	1.121	-0.333
(B) (a)	0.965	1.075	-0.096
(B) (b)	0.956	1.132	-0.395

Table S17. Calculated shielding values of the reference TMS molecule: (A) at the GIAO/B3LYP/6-311+G(2d,p)/CPCM level with energy minimization in the gas using a variety of functionals and basis sets, and (B) at the GIAO with the same level of theory as geometry optimization.

Minimization Method (Gas Phase)	(A)		(B)	
	($CPCM/CHCl_3$)	($CPCM=CCl_4$)	($CPCM/CHCl_3$)	($CPCM=CCl_4$)
B3LYP/6-31+G(d)	31.79	31.79	32.07	32.07
B3LYP/6-311++G(d,p)	31.90	31.90	31.97	31.97
B3LYP-D3/6-31+G(d)	31.81	31.81	32.08	32.08
B3LYP-D3/6-311++G(d,p)	31.91	31.91	31.99	31.99
M06-2X/6-31+G(d)	31.87	31.86	32.18	32.18
M06-2X /6-311++G(d,p)	31.96	31.96	32.08	32.08
APFD/6-31+G(d)	31.83	31.83	32.01	32.01
APFD/6-311++G(d,p)	31.93	31.91	31.83	31.83
ωB97XD/6-31+G(d)	31.84	31.84	32.12	32.12
ωB97XD /6-311++G(d,p)	31.93	31.93	31.96	31.96
PBE0/6-31+G(d)	31.83	31.83	32.08	31.99
PBE0/6-311++G(d,p)	31.91	31.91	31.80	31.80
